# Thermal Rearrangement of 5-(2-Hydroxy-6-oxocyclohexyl)-5*H*-chromeno[2,3-*b*]pyridines

**DOI:** 10.3390/molecules28073139

**Published:** 2023-03-31

**Authors:** Yuliya E. Ryzhkova, Fedor V. Ryzhkov, Michail N. Elinson, Anatoly N. Vereshchagin, Roman A. Novikov, Artem N. Fakhrutdinov

**Affiliations:** N. D. Zelinsky Institute of Organic Chemistry Russian Academy of Sciences, 47 Leninsky Prospekt, 119991 Moscow, Russia; yu_ryzhkova@ioc.ac.ru (Y.E.R.); ryzhkovfv@ioc.ac.ru (F.V.R.); vereshchagin@ioc.ac.ru (A.N.V.); novikovfff@bk.ru (R.A.N.); fak.part@gmail.com (A.N.F.)

**Keywords:** rearrangement, NMR study, chromeno[2,3-*b*]pyridine, dimethyl sulfoxide, 2,3,4,9-tetrahydro-1*H*-xanthene, 5,6,7,8,9,10-hexahydrobenzo[*b*][1,6]naphthyridine

## Abstract

Some of the most important transformations in organic chemistry are rearrangement reactions, which play a crucial role in increasing synthetic efficiency and molecular complexity. The development of synthetic strategies involving rearrangement reactions, which can accomplish synthetic goals in a very efficient manner, has been an evergreen topic in the synthetic chemistry community. Xanthenes, pyridin-2(1*H*)-ones, and 1,6-naphthyridines have a wide range of biological activities. In this work, we propose the thermal rearrangement of 7,9-dihalogen-substituted 5-(2-hydroxy-6-oxocyclohexyl)-5*H*-chromeno[2,3-*b*]pyridines in DMSO. Previously unknown 5,7-dihalogenated 5-(2,3,4,9-tetrahydro-1*H*-xanthen-9-yl)-6-oxo-1,6-dihydropyridines and 10-(3,5-dihalogen-2-hydroxyphenyl)-5,6,7,8,9,10-hexahydrobenzo[*b*][1,6]naphthyridines were synthesized with excellent yields (90–99%). The investigation of the transformation using ^1^H-NMR monitoring made it possible to confirm the ANRORC mechanism. The structures of synthesized compounds were confirmed by 2D-NMR spectroscopy.

## 1. Introduction

Some of the most important transformations in organic chemistry are rearrangement reactions, which play a crucial role in increasing synthetic efficiency and molecular diversity [1]. The concomitant cleavage and reconstruction of chemical bonds to form new useful molecules is a process of remarkable complexity in synthetic organic chemistry. Two significant scientific topics in synthetic chemistry are the highly effective formation of carbon–carbon bonds and the building of corresponding molecular skeletons [2]. As a result, the development of synthetic strategies involving rearrangement reactions that can effectively accomplish these two synthetic goals in a very efficient manner is still an evergreen topic in the synthetic chemistry community.

There is a plethora of rearrangement reactions that have been developed and applied in organic chemistry. They have different mechanisms and drastically different reaction conditions. They include the Wagner–Meerwein, Tiffeneau–Demjanov, Beckman, Baeyer–Villiger, Claisen, Wolf, and Pinacol reactions, amongst others [3,4,5,6,7,8,9,10,11,12,13,14,15,16,17,18,19].

ANRORC rearrangement occurs in the nucleophilic substitution of heterocyclic compounds and represents a process of the subsequent Addition of the Nucleophile, Ring Opening, and Ring Closure in the same molecule [20,21]. These rearrangements have been studied extensively [22]. Many heterocyclic rearrangements are known: aminoimidazoles [23], oxadiazoles [24,25,26], triazole-carboxamides [27], and imidazo[1,4]thiazine [28].

Transformations of chromeno[2,3-*b*]pyridines are quite common in the literature. However, these are typically just modifications of functional groups [29,30]. The most interesting reactions are those that are caused by the formation of entirely new compounds. However, publications about these are scarce. Among them are ones about rearrangement [31] and cyclization [32].

**Figure 1:**
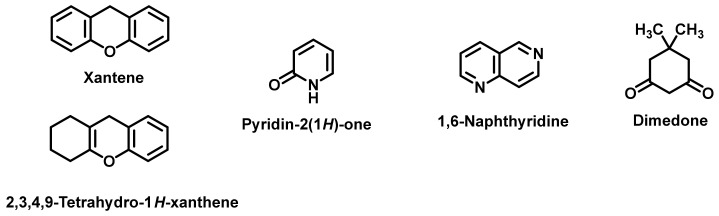
Pharmacophore fragments of biologically active compounds.

Xanthenes (Figure 1) exhibit these types of pharmacological activities such as antiproliferative [33], antibacterial [34], and antioxidant [35] activities; additionally, they are able to inhibit neuropeptide receptors, which have considerable therapeutic benefits for treating obesity [36].

Pyridin-2(1*H*)-ones (Figure 1) exhibit antiproliferative properties [37], and they also increase the body’s resistance to oxidative stress [38].

Derivatives of 1,6-Naphthyridine (Figure 1) act as anticancer [39], antiviral [40], antiasthmatic [41], anticonvulsant [42], and analgesic [43] agents.

The dimedone fragment (Figure 1) can be found in numerous compounds that are effective in treating a variety of disorders, such as tropical infectious diseases [44]. Dimedone and its derivatives have shown numerous biological properties, including antibacterial [45,46], antifungal [46], and antioxidant [47] properties.

Finally, the straightforward and simple synthesis of novel complex compounds that are valuable from the perspective of biological activity is a relevant goal in organic chemistry.

## 2. Results and Discussion

**Scheme 1:**
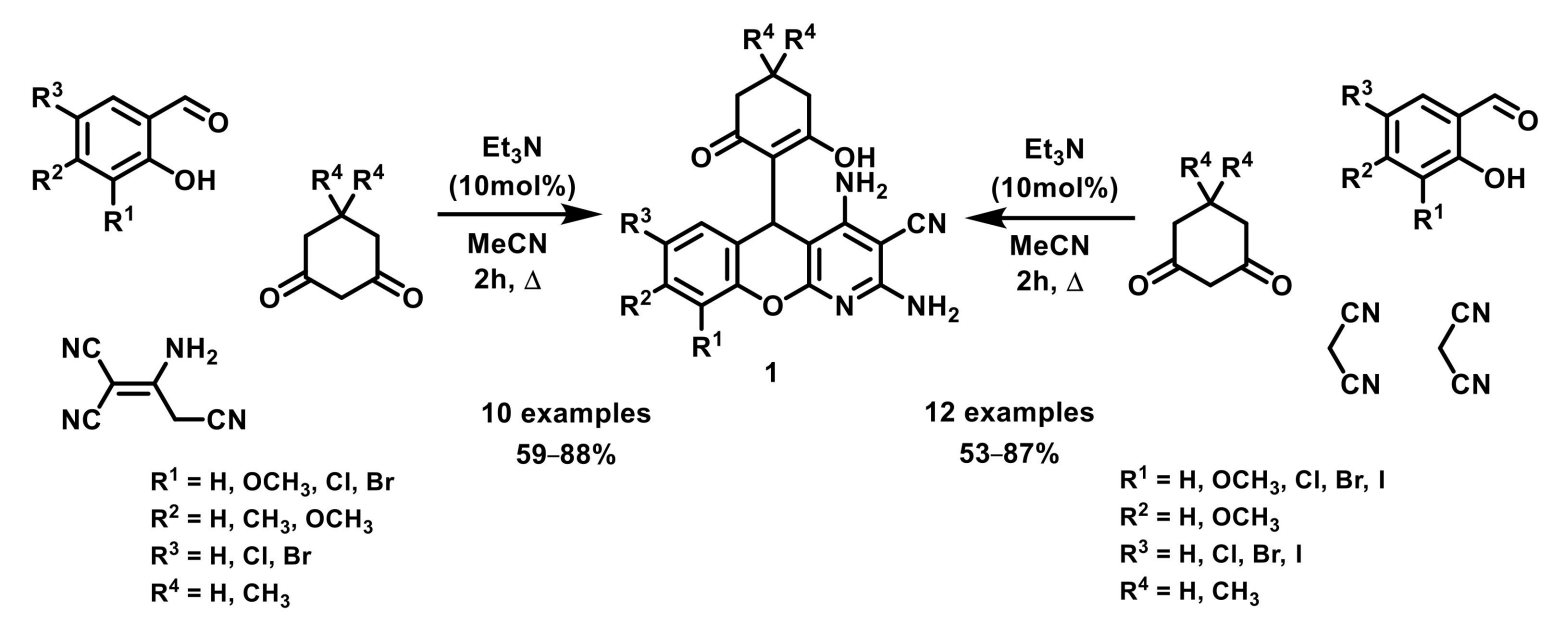
Multicomponent synthesis of 5-(2-hydroxy-6-oxocyclohexyl)-5*H*-chromeno[2,3-*b*]pyridines **1**.

Previously, our scientific group synthesized a wide variety of chromeno[2,3-*b*]pyridines with various structures [48,49,50,51,52,53]. In particular, 5-(2-hydroxy-6-oxocyclohexyl)-5*H*-chromeno[2,3-*b*]pyridines **1** were synthesized by two methods (Scheme 1): using three- [54] and pseudo-four-component reactions [55].

### 2.1. Thermal Rearrangement of 5-(2-Hydroxy-6-oxocyclohexyl)-5H-chromeno[2,3-b]pyridines

This rearrangement was discovered during the development of the approach to 5-(2-hydroxy-6-oxocyclohexyl)-5*H*-chromeno[2,3-*b*]pyridines **1** (Scheme 1) [54,55]. It has been found that 5-(2,3,4,9-tetrahydro-1*H*-xanthen-9-yl)-6-oxo-1,6-dihydropyridine **2a** appears in the ^1^H-NMR spectrum after standing dichloro-substituted compound **1** in an NMR tube with DMSO-*d*_6_ for one week.

**Scheme 2:**
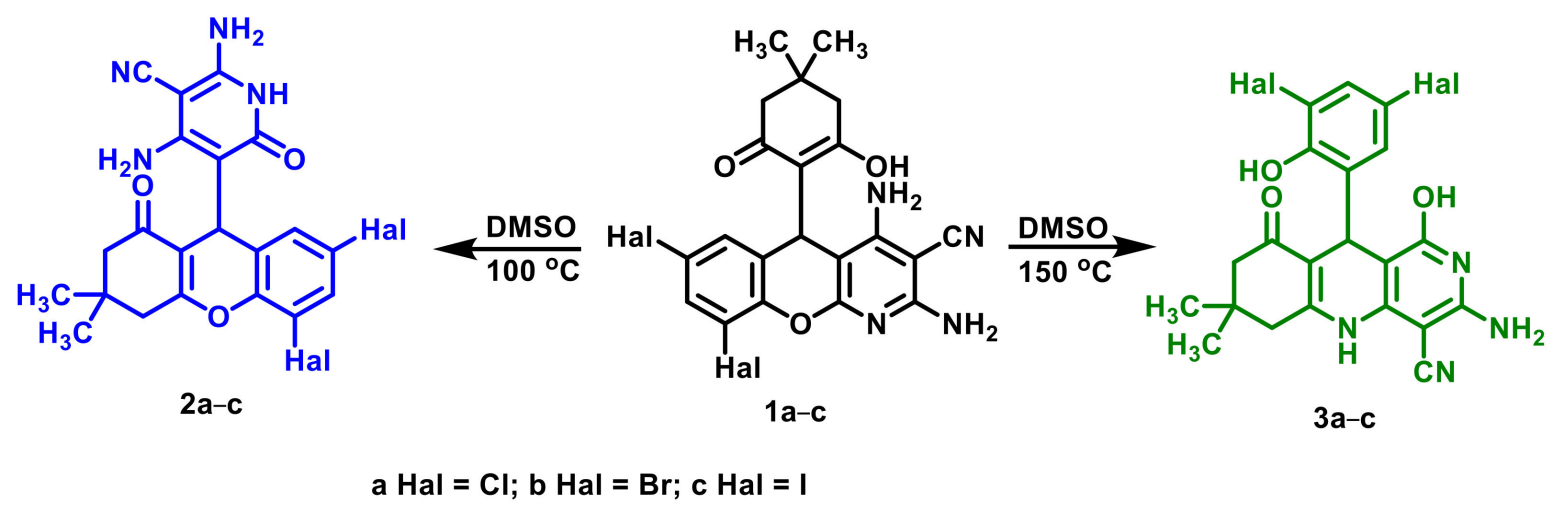
Thermal rearrangement of 7,9-dihalogen-substituted 5-(2-hydroxy-6-oxocyclohexyl)-5*H*-chromeno[2,3-*b*]pyridines **1**.

Individual experiments revealed that depending on the temperature conditions, 7,9-dihalogen-substituted chromeno[2,3-*b*]pyridines **1a–c** are converted into the corresponding 5,7-dihalogenated 5-(2,3,4,9-tetrahydro-1*H*-xanthen-9-yl)-6-oxo-1,6-dihydropyridines **2a–c** and 10-(3,5-dihalogen-2-hydroxyphenyl)-5,6,7,8,9,10-hexahydrobenzo-[*b*][1,6]naphthyridines **3a–c** (Scheme 2).

Regarding the example of compound **1a**, optimal rearrangement conditions were found (Table 1). First of all, the reaction was studied in DMSO and DMF. After chromeno[2,3-*b*]pyridine **1a** was heated in DMSO at 100 °C for 1 h, 5-(2,3,4,9-tetrahydro-1*H*-xanthen-9-yl)-6-oxo-1,6-dihydropyridine **2a** was obtained, with 99% yield (Table 1, Entry 1).

Heating at 120 °C resulted in a mixture of compounds **2a** and **3a** in a ratio of 9:1 (Table 1, Entry 2). After heating chromeno[2,3-*b*]pyridine **1a** at 150 °C in DMSO, 5,6,7,8,9,10-Hexahydrobenzo[*b*][1,6]naphthyridine **3a** was isolated, with a yield of 98% (Table 1, Entry 3).   Slightly lower yields of target compounds **2a** and **3a** were achieved in DMF (Table 1, Entries 4 and 5).

Heating 5-(2-hydroxy-6-oxocyclohexyl)-5*H*-chromeno[2,3-*b*]pyridine **1a** in water, acetonitrile, *n*-propanol, and dioxane did not show good results (Table 1, Entries 6–9).  In these reactions, mixtures of starting compound **1a**, the target 5-(2,3,4,9-tetrahydro-1*H*-xanthen-9-yl)-6-oxo-1,6-dihydropyridine **2a,** and 5,6,7,8,9,10-hexahydrobenzo[b][1,6]naphthyridine **3a** were mainly isolated. Reducing the reaction time in the best solvent, DMSO, resulted in a decrease in the yields of the compounds **2a** and **3a** (Table 1, Entries 10 and 11).

Under these found optimal conditions, the thermal rearrangement of 7,9-dihalogen-substituted 5-(2-hydroxy-6-oxocyclohexyl)-5*H*-chromeno[2,3-*b*]pyridines **1a–c** leads to 5,7-dihalogenated 5-(2,3,4,9-tetrahydro-1*H*-xanthen-9-yl)-6-oxo-1,6-dihydropyridines **2a–c** and **3a–c** with 90–99% yields after 1 h (Table 2).

By using ^1^H and ^13^C NMR data (see Supplementary Materials), IR spectroscopy, mass spectrometry, and elemental analysis, the structures of the obtained compounds, **2a–c** and **3a–c,** were confirmed. Additionally, two-dimensional (2D) NMR spectroscopy methods were used to carry out structure investigations for compounds **2a** and **3a** (see Section 2.3 and Supplementary Materials).

After the reaction in DMSO had completed, water was added to the reaction mixture, and target compounds **2** or **3** crystallized in a pure form without the need for chromatographic purification or additional recrystallization. Thermal rearrangement is easy to perform and only requires the use of basic equipment.

**Scheme 3:**
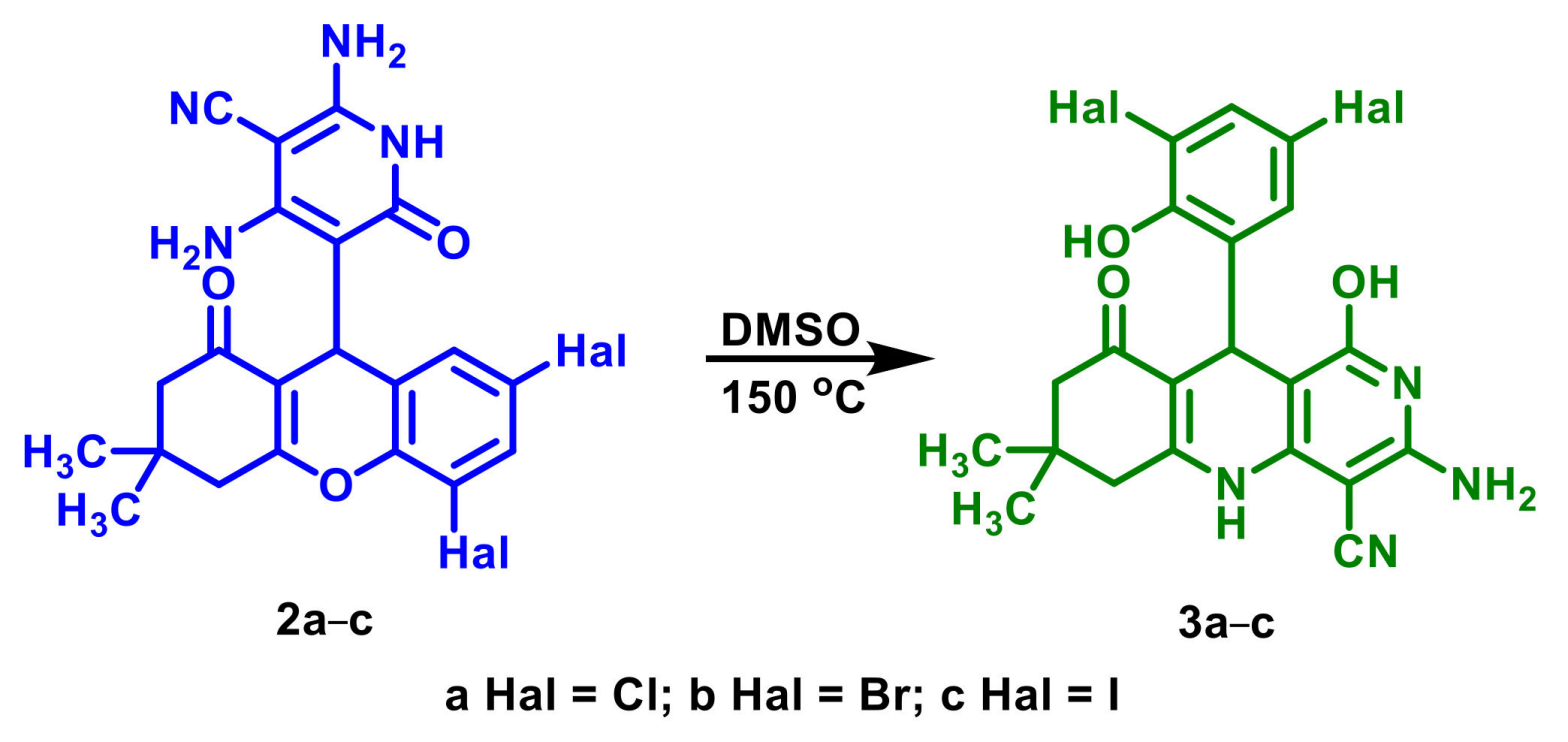
Thermal rearrangement of 5,7-dihalogenated 5-(2,3,4,9-tetrahydro-1*H*-xanthen-9-yl)-6-oxo-1,6-dihydropyridines **2**.

Additionally, it was found that isolated 5,7-dihalogenated 5-(2,3,4,9-tetrahydro-1*H*-xanthen-9-yl)-6-oxo-1,6-dihydropyridines, **2a–c,** are able to be converted into 10-(3,5-dihalogen-2-hydroxyphenyl)-5,6,7,8,9,10-hexahydrobenzo[*b*][1,6]naphthyridines, **3a–c,** with 93–97% yields when they are heated up to 150 °C in DMSO for 1 h (Scheme 3).

**Figure 2:**
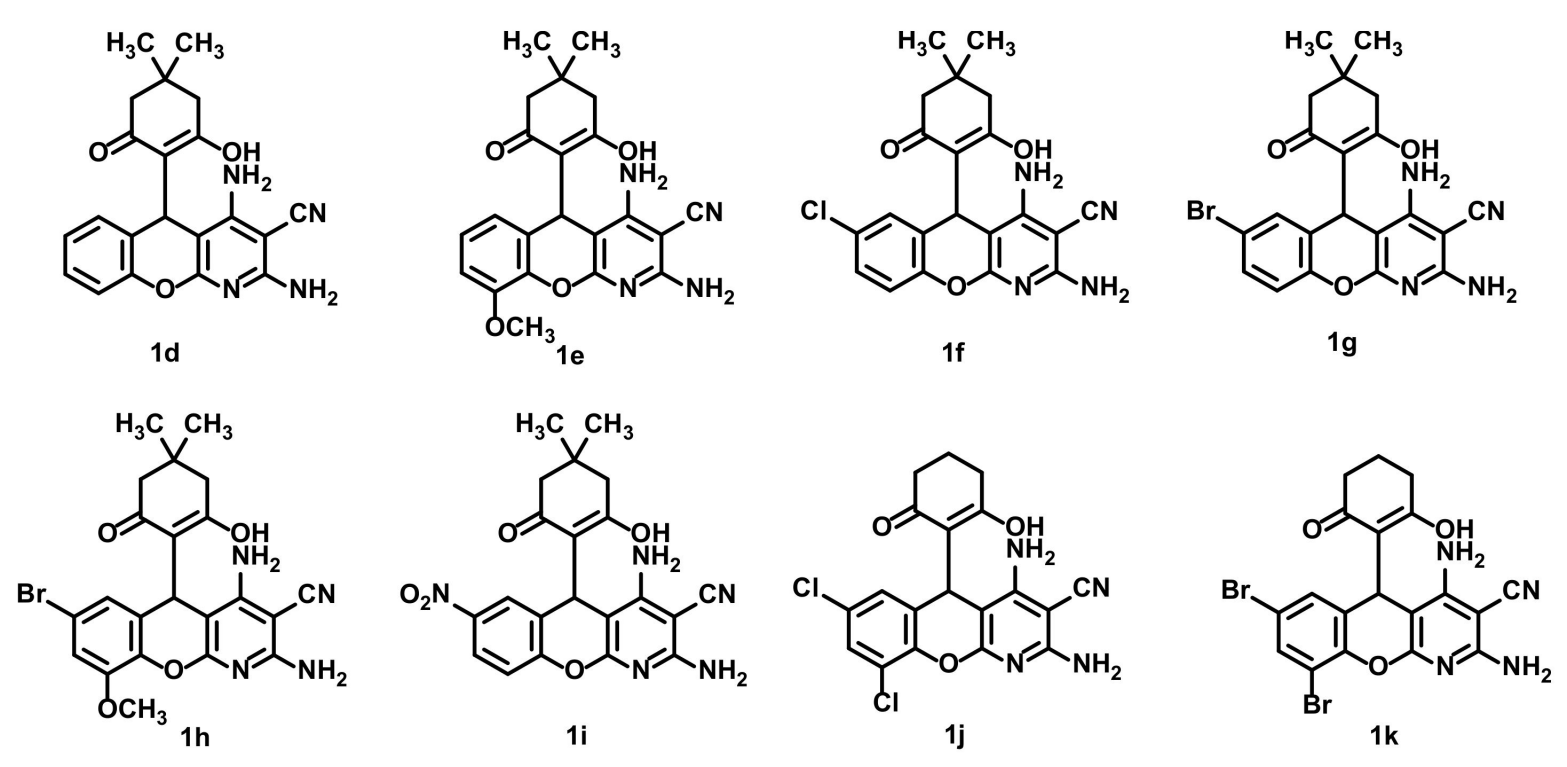
Other 5-(2-hydroxy-6-oxocyclohexyl)-5*H*-chromeno[2,3-*b*]pyridines **1** studied as objects of thermal rearrangement.

Further, the generality of this thermal rearrangement was investigated (Figure 2, Table 3). Upon heating unsubstituted chromeno[2,3-*b*]pyridine **1d**, as well as 9-methoxy- and 7-bromo-9-methoxy-substituted compounds **1e** and **1h** at 100 °C, the starting compound was isolated (Table 3, Entries 1, 2, and 5). The heating of chromeno[2,3-*b*]pyridines **1d**, **1e,** and **1h** at 150 °C for 1 h resulted in the decomposition of initial structure **1** (Table 3, Entries 1, 2, and 5).

Involving monohalogenated compounds **1f** and **1g** in the rearrangement showed different results (Table 3, Entries 3 and 4). The rearrangement proceeded partly at 100 °C, but at 150 °C, it resulted in the formation of compounds **2** and **3**, as well as a partial decomposition of starting chromeno[2,3-*b*]pyridines **1f** and **1g**.

The rearrangement of chromeno[2,3-*b*]pyridine **1i** is also possible. It is supposed that a strong acceptor in molecule **1,** in this case, a nitro group in the seventh position, makes the rearrangement more favorable. The rearrangement takes place even during the synthesis of starting chromeno[2,3-*b*]pyridine **1i**. According to the ^1^H NMR spectra, all three compounds (**1i**, **2i,** and **3i**) were detected at once in a ratio of approximately 1:1:1 in the reaction mixture after the synthesis of chromeno[2,3-*b*]pyridine **1i**.

In the case of 1,3-cyclohexanedione derivatives **1j** and **1k**, the rearrangement also take place with 78–82% yields (Table 3, Entries 6 and 7). At both temperatures, however, it is accompanied by the partial decomposition of the starting dihalogen-substituted chromeno[2,3-*b*]pyridines, **1j** and **1k**. Compounds **2j,k** and **3j,k** could not be isolated in pure forms, even after several recrystallizations.

**Scheme 4:**
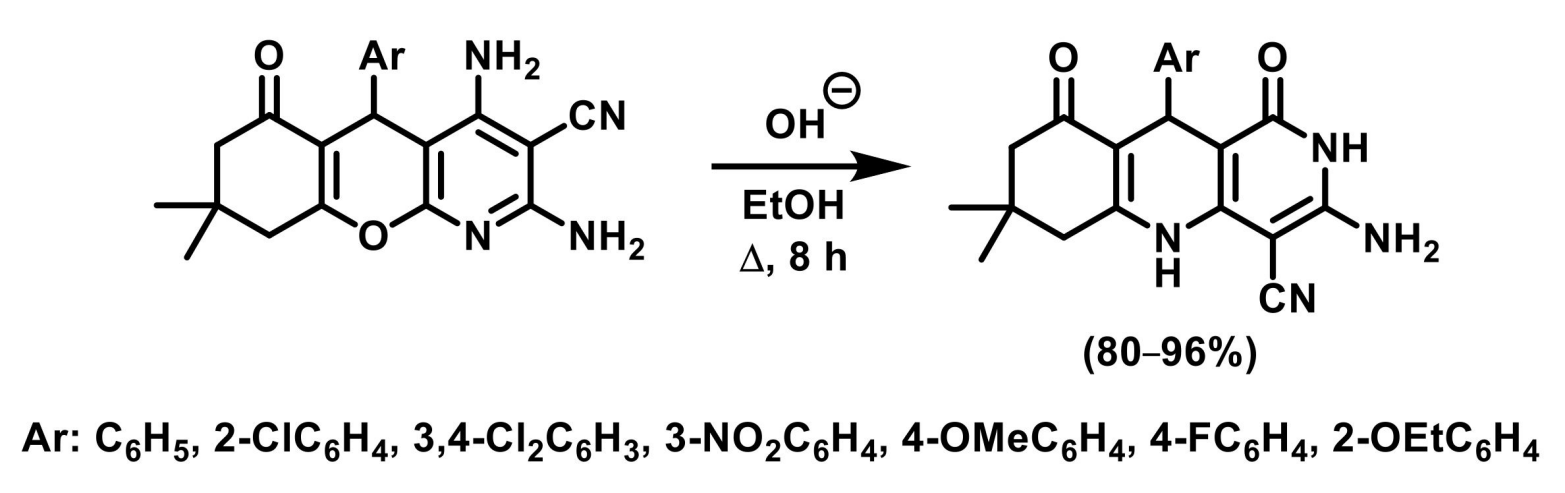
ANRORC rearrangement of 6,7,8,9-tetrahydro-5*H*-chromeno[2,3-*b*]pyridines.

Previously, the synthesis of benzo[*b*][1,6]naphthyridine derivatives in a basic medium via the rearrangement of 6,7,8,9,10-tetrahydro-5*H*-chromeno[2,3-*b*]pyridines has been described (Scheme 4) [22]. However, for our compounds, **1**, these conditions were not suitable. Chromeno[2,3-*b*]pyridines **1d**, **1e,** and **1g** remained unchanged, and upon trying to introduce chromeno [2,3-*b*]pyridines **1a–c**,**f**,**g**,**i–k** into the reaction, only mixtures of compounds **2** and **3** formed in various ratios.

### 2.2. The ^1^H-NMR Monitoring of Thermal Rearrangement

**Scheme 5:**
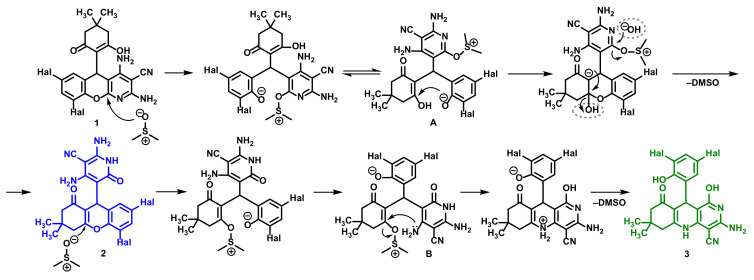
Proposed mechanism of thermal rearrangement of 5-(2-hydroxy-6-oxocyclohexyl)-5*H*-chromeno[2,3-*b*]pyridines **1**.

The following ANRORC (Addition of the Nucleophile, Ring Opening, and Ring Closure) mechanism of the thermal rearrangement of chromeno[2,3-*b*]pyridines **1** into 5,7-dihalogenated 5-(2,3,4,9-tetrahydro-1*H*-xanthen-9-yl)-6-oxo-1,6-dihydropyridines **2** and 10-(3,5-dihalogen-2-hydroxyphenyl)-5,6,7,8,9,10-hexahydrobenzo[b][1,6]naphthy-ridines **3** (Scheme 5) was proposed based on the literature data [21,31].

At the first stage, a nucleophilic attack of the DMSO molecule at the pyridine fragment of chromeno[2,3-*b*]pyridine **1** takes place. This results in the opening of the pyran ring. The intramolecular interaction of the oxygen anion of the dihalogen-substituted benzene fragment with the electron-deficient carbon atom of cyclohexanedione leads to the formation of another pyran ring (ring closure). Additionally, 5-(2,3,4,9-tetrahydro-1*H*-xanthen-9-yl)-6-oxo-1,6-dihydropyridine **2** (Scheme 5) is formed with the expulsion of the DMSO molecule. The first stage occurs regardless of whether the process is carried out at 100 °C or 150 °C.

Further, if the process is carried out at 150 °C, compound **2** is also attacked by the DMSO molecule and undergoes pyran ring opening. Then, the amino group of pyridinone participates in cyclization (ring closure) to form a dihydropyridine fragment of the final structure. Further transformations form 5,6,7,8,9,10-hexahydrobenzo[b][1,6]naphthyridine **3** (Scheme 5).

**Figure 3:**
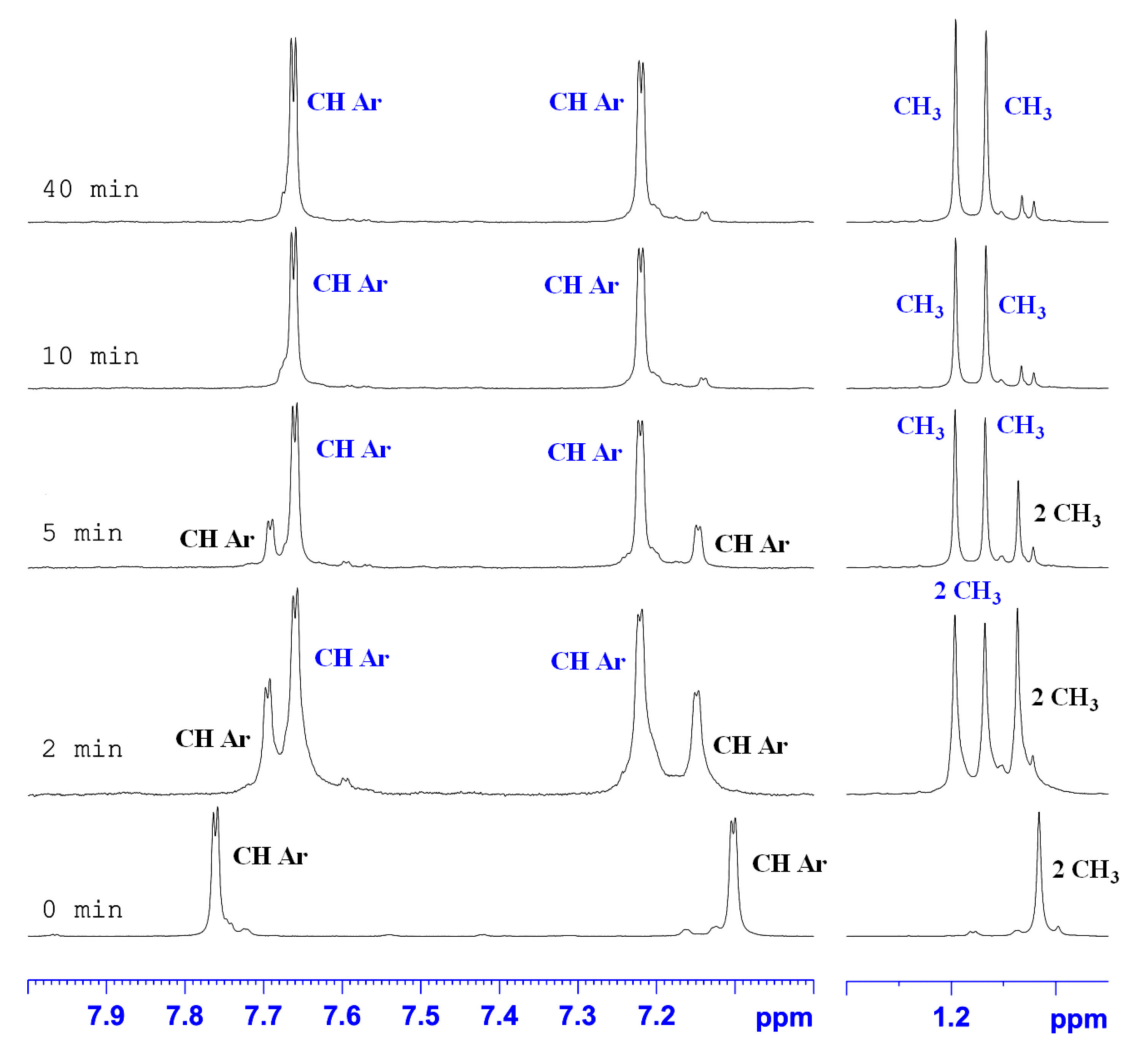
Monitoring of the rearrangement process at 100 °C by ^1^H-NMR (for compound **1b**). Black captions used for **1b**; blue captions used for **2b**.

**Figure 4:**
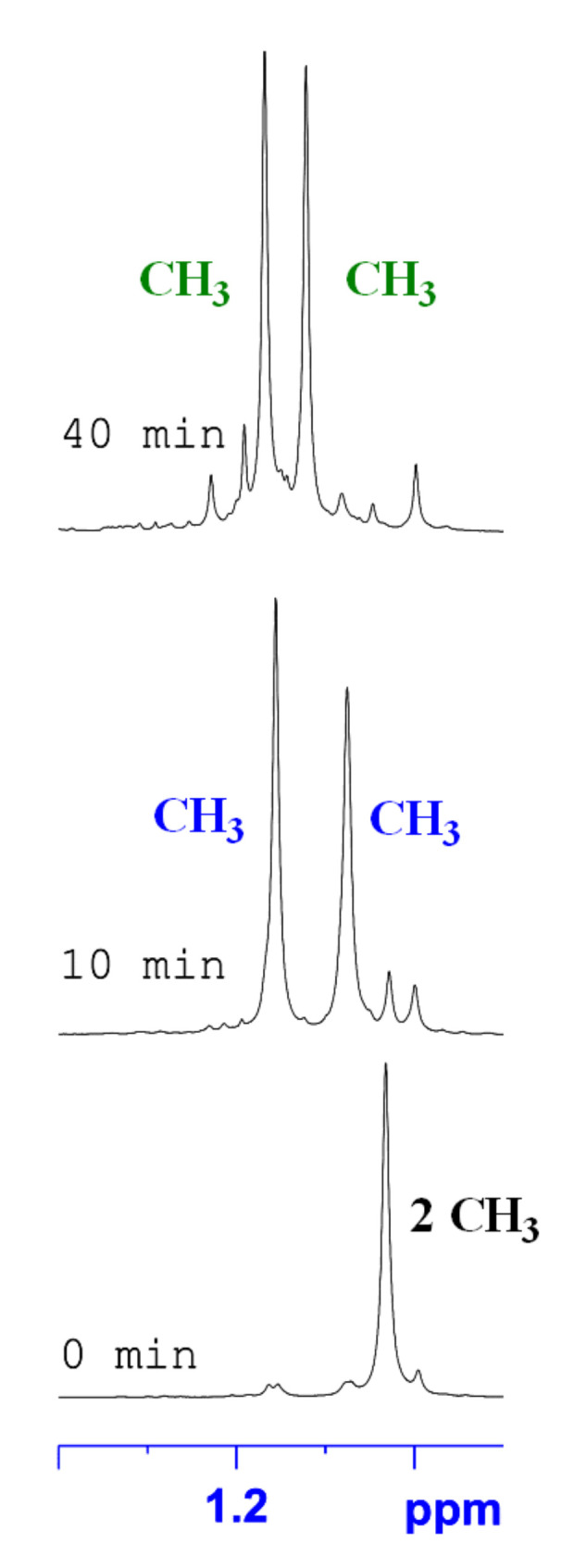
Monitoring of the rearrangement process at 150 °C by ^1^H-NMR (for compound **1b**).

This rearrangement process was investigated using ^1^H-NMR monitoring (Figure 3 and Figure 4). The experiments were carried out in NMR tubes with dilute solutions (15 mg of substance per 600 μL of DMSO-*d*_6_).

The first stage involves investigating the transformation of chromeno[2,3-*b*]pyridine **1b** at 100 °C. Figure 3 shows the change in intensity of signals from the protons of methyl groups and aromatic protons in the ^1^H-NMR spectra. The process proceeds rapidly; 2 min after the start of the heating phase, 5-(2,3,4,9-tetrahydro-1*H*-xanthen-9-yl)-6-oxo-1,6-dihydropyridine **2b** begins to prevail over starting compound **1b**. chromeno[2,3-*b*]pyridine **1b** almost completely converts to 2,3,4,9-tetrahydro-1*H*-xanthene **2b** after 10 min.

At the next stage, we studied the transformation of chromeno[2,3-*b*]pyridine **1b** at 150 °C. Figure 4 shows the change in the intensity of signals from the protons of methyl groups in the ^1^H-NMR spectra. This rearrangement also proceeds rapidly. After 10 min, 2,3,4,9-tetrahydro-1*H*-xanthene **2b** is mostly converted to 5,6,7,8,9,10-hexahydrobenzo[*b*][1,6]naphthyridines **3b**.

Therefore, the thermal rearrangement mechanism proposed above (Scheme 4) does not contradict the monitoring data.

### 2.3. Confirmation of the Structure of the Synthesized 2,3,4,9-Tetrahydro-1H-xanthenes **2** and 5,6,7,8,9,10-Hexahydrobenzo[b][1,6]naphthyridines **3**

**Figure 5:**
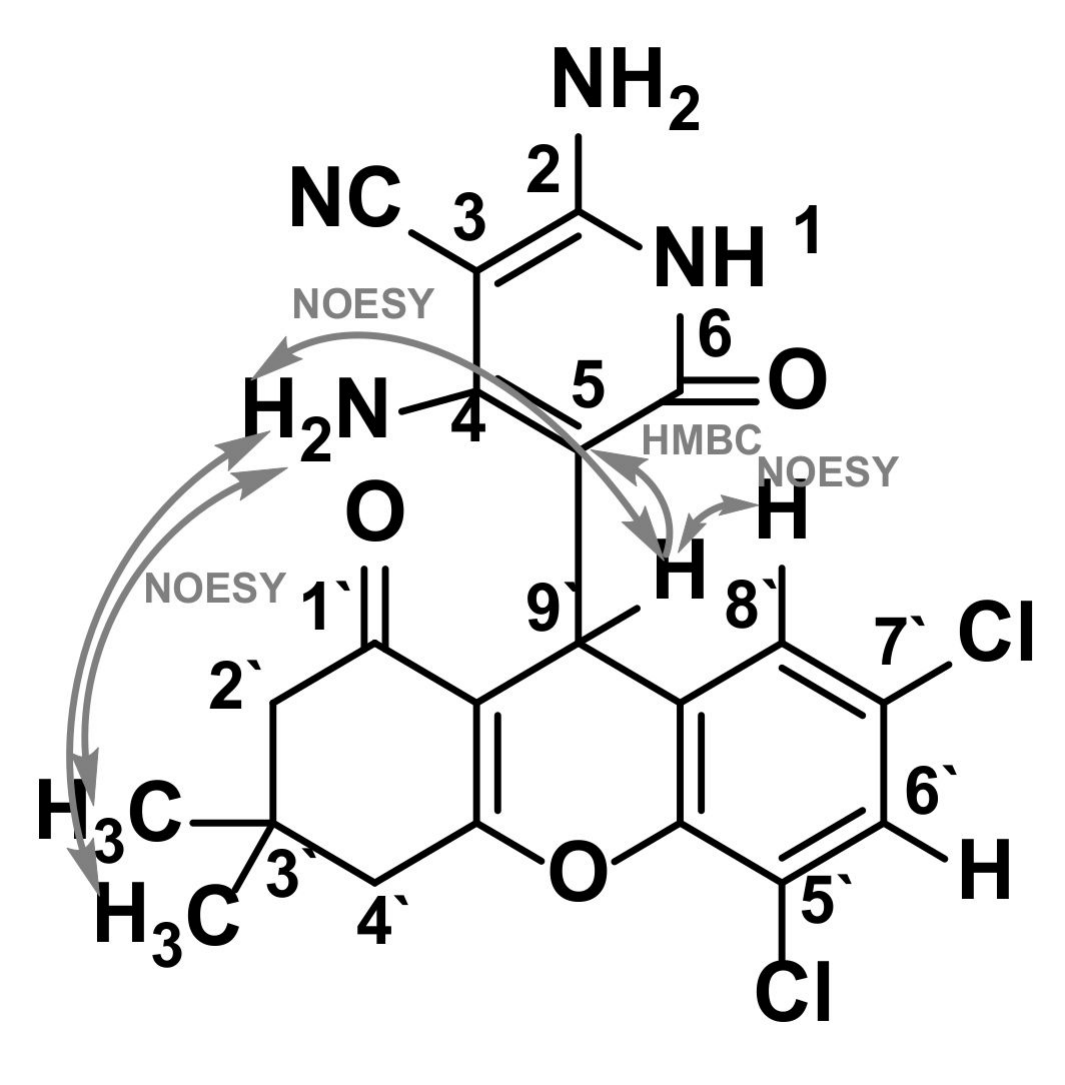
The structure of 5-(2,3,4,9-tetrahydro-1*H*-xanthen-9-yl)-6-oxo-1,6-dihydropyridine **2a**. Key 2D NMR correlations are shown by arrows.

**Figure 6:**
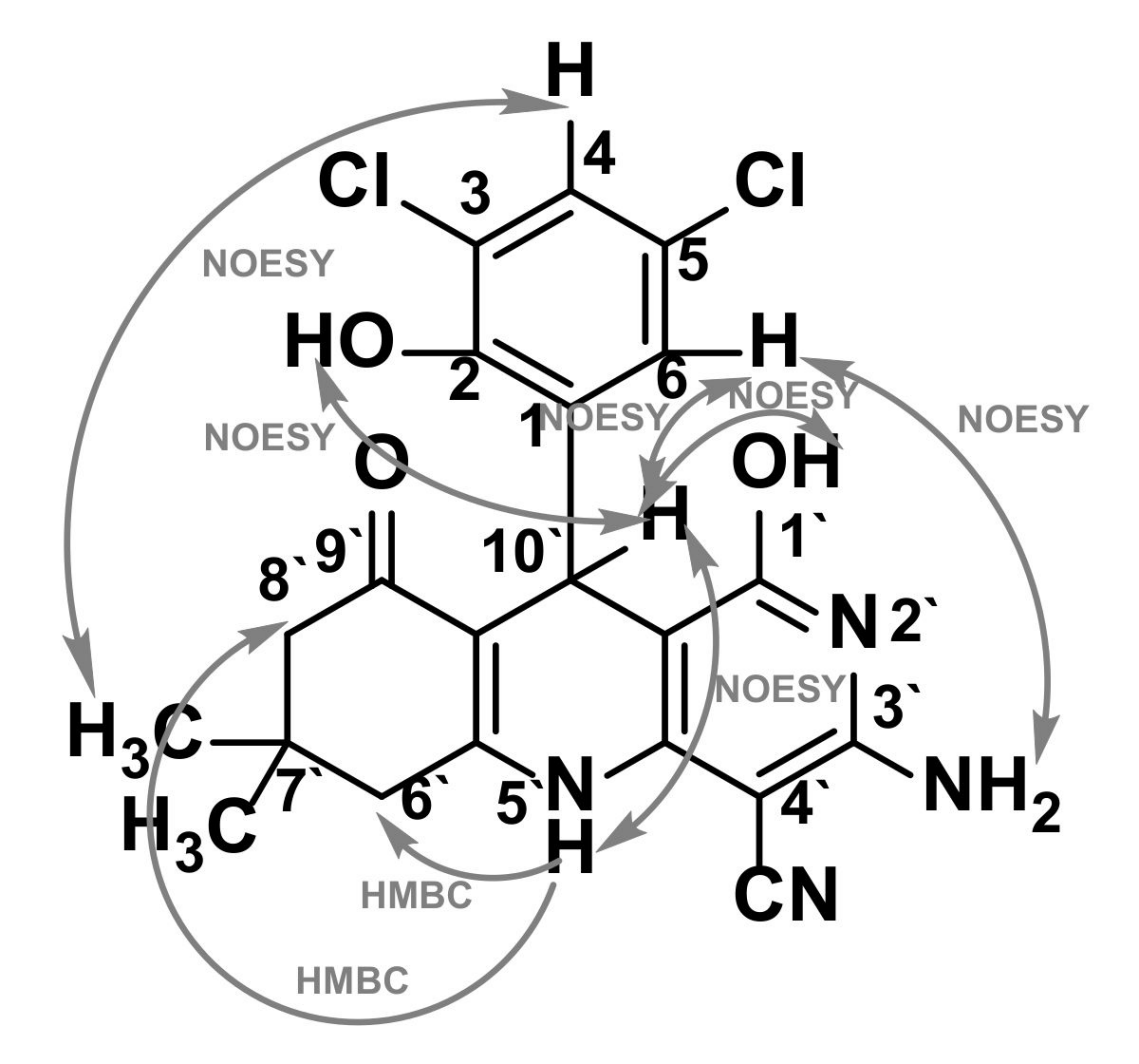
The structure 10-(3,5-dichloro-2-hydroxyphenyl)-5,6,7,8,9,10-hexahydrobenzo[*b*][1,6]-naphthyridines **3a**. Key 2D NMR correlations are shown by arrows.

The structures of compounds **2a** and **3a** were additionally confirmed by ^1^H, ^13^C, and ^15^N NMR spectroscopy, including two-dimensional (2D) methods (see Supplementary Information). Key correlation interactions are shown by the arrows in Figure 5 and Figure 6.

First, 5-(2,3,4,9-Tetrahydro-1*H*-xanthen-9-yl)-6-oxo-1,6-dihydropyridine **2a** (Figure 5) was considered. From the {^1^H-^15^N}-HSQC spectrum, it was revealed that the molecule contains two NH_2_ groups and one NH group. From the {^1^H-^1^H}-NOESY spectrum, the following NOE interactions were revealed: one between the methyl groups and an NH_2_ group at C^4^; one between a proton at C^9^′ and an NH_2_ group at C^4^; one between the protons at C^9^′ and C^8^′. It should be especially noted that there are no correlations between the cyclohexanedione and the condensed benzene fragment; therefore, they are in the same plane and form a tricyclic system.

In addition, no interactions between the pyridinone ring and the xanthene system were recorded in the {^1^H-^13^C} HMBC spectrum.

Based on the results obtained, as well as the data from IR spectroscopy and mass spectrometry, it can be concluded that the proposed structure for compound **2a** is correct.

Next, 10-(3,5-dichloro-2-hydroxyphenyl)-5,6,7,8,9,10-hexahydrobenzo[b][1,6]-naphthyridine **3a** was considered (Figure 6). From the ^15^N- and {^1^H-^15^N}-HSQC spectra, it was revealed that the molecule contains one NH_2_ group and one NH group. From the {^1^H-^1^H}-NOESY spectrum, the following NOE interactions were revealed: one between the methyl groups and an aromatic proton at C^4^; one between an aromatic proton at C^6^ and an NH_2_ group; one between a proton at C^10^′ and hydroxy groups, as well as N(5′)H. It should be especially noted that there are no correlations between the cyclohexanedione and pyridine fragments; therefore, they are in the same plane and form a tricyclic system.

A tricyclic system was also confirmed by the {^1^H-^13^C} HMBC spectrum. Cross peaks were found for aliphatic NH and CH_2_. In addition, no interactions between the dihalogen-substituted benzene fragment and the naphthyridine system were detected.

Based on the results obtained, as well as the data from IR spectroscopy and mass spectrometry, it can be concluded that the proposed structure for compound **3a** is correct.

The 2D NMR spectra of the compounds **2a** and **3a** are presented in the Supplementary Materials.

## 3. Materials and Methods

### 3.1. General Methods

Solvents were purchased from commercial suppliers and used as received, without purification. The synthesis of 5-(2-Hydroxy-6-oxocyclohexyl)-5*H*-chromeno[2,3-*b*]pyridine-3-carbonitriles **1** was performed in accordance with the following methods [54,55].

Using Gallenkamp melting-point apparatus (Gallenkamp & Co., Ltd., London, UK), melting points were measured. At room temperature, ^1^H and ^13^C-NMR spectra were obtained in DMSO-*d*_6_ with a Bruker AM300 spectrometer (Bruker Corporation, Billerica, MA, USA). The values for chemical shift are given in relation to Me_4_Si. A Bruker AV500 spectrometer (Bruker Corporation, Billerica, MA, USA) was used to record two-dimensional (2D) NMR spectra. A Bruker AV400 spectrometer (Bruker Corporation, Billerica, MA, USA) was used to register the ^1^H-NMR monitoring spectra. IR spectra were determined with a Bruker ALPHA-T FT-IR spectrometer (Bruker Corporation, Billerica, MA, USA) in KBr pellets. With a Kratos MS-30 spectrometer (Kratos Analytical Ltd., Manchester, UK), mass spectra (EI = 70 eV) were acquired. For elemental analysis, a 2400 Elemental Analyzer (Perkin Elmer Inc., Waltham, MA, USA) was applied.

### 3.2. Thermal Rearrangement of 7,9-Dihalogenated 5-(2-Hydroxy-6-oxocyclohexyl)-5H-chromeno[2,3-b]pyridine-3-carbonitriles **1** at 100 °C

A solution of 7,9-dihalogenated 5-(2-hydroxy-6-oxocyclohexyl)-5*H*-chromeno[2,3-*b*]-pyridine-3-carbonitrile **1** (0.5 mmol) was stirred in DMSO (0.5 mL) for 1 h at 100 °C. Upon completion of the reaction, the reaction mixture was allowed to cool to room temperature, water (5 mL) was added to the reaction mixture, and the resulting precipitate of pure 5,7-dihalogenated 5-(1-oxo-2,3,4,9-tetrahydro-1*H*-xanthen-9-yl)-6-oxo-1,6-dihyd-ropyridine-3-carbonitrile **2** was separated by filtration, washed with cold ethanol (2 × 3 mL), and dried.

***2,4-Diamino-5-(5,7-dichloro-3,3-dimethyl-1-oxo-2,3,4,9-tetrahydro-1H-xanthen-9-yl)-6-oxo-1,6-dihydropyridine-3-carbonitrile (2a):*** Yellowish solid; yield—99% (0.220 g); m.p. = 296–297 °C (decomp.) (from DMSO-H_2_O); FTIR (KBr) cm^−1^: 3214, 2952, 2203, 1655, 1620, 1573, 1458, 1376, 1242, and 1034. ^1^H-NMR (300 MHz, DMSO-*d*_6_): *δ* 0.98 (s, 3H, CH_3_), 1.06 (s, 3H, CH_3_), 2.09 (d, ^2^*J* = 16.2 Hz, 1H, CH_2_), 2.27 (d, ^2^*J* = 16.2 Hz, 1H, CH_2_), 2.44 (d, ^2^*J* = 16.2 Hz, 1H, CH_2_), 2.59 (d, ^2^*J* = 16.2 Hz, 1H, CH_2_), 4.89 (s, 1H, CH), 6.34 (br s, 2H, NH_2_), 6.54 (br s, 2H, NH_2_), 6.92 (s, 1H, CH Ar), 7.43 (s, 1H, CH Ar), and 9.67 (br s, 1H, NH) ppm; ^13^C-NMR (75 MHz, DMSO-*d*_6_): *δ* 26.4, 27.5, 28.9, 31.8, 40.3, 50.3, 62.1, 98.2, 110.2, 116.8, 120.4, 126.8 (2C), 126.9, 129.4, 145.2, 153.0, 154.6, 159.8, 164.4, and 196.4 ppm; MS (*m*/*z*, relative intensity %): 448 (^37^Cl, ^37^Cl, [M]^+^, 1), 446 (^37^Cl, ^35^Cl, [M]^+^, 6), 444 (^35^Cl, ^35^Cl, [M]^+^, 9), 364 (^37^Cl, ^37^Cl, 3), 362 (^37^Cl, ^35^Cl, 18), 360 (^35^Cl, ^35^Cl, 24), 283 (11), 279 (7), 243 (^37^Cl, ^37^Cl, 1), 241 (^37^Cl, ^35^Cl, 5), 239 (^35^Cl, ^35^Cl, 8), 166 (11), 150 (17), 122 (27), 77 (39), and 43 (100); Anal. calcd. for C_21_H_18_Cl_2_N_4_O_3_: C, 56.64; H, 4.07; N, 12.58%; found: C, 56.57; H, 4.12; N, 12.54%.

***2,4-Diamino-5-(5,7-dibromo-3,3-dimethyl-1-oxo-2,3,4,9-tetrahydro-1H-xanthen-9-yl)-6-oxo-1,6-dihydropyridine-3-carbonitrile (2b):*** Yellowish solid; yield—98% (0.262 g); m.p. = 294–295 °C (decomp.) (from DMSO-H_2_O); FTIR (KBr) cm^−1^: 3330, 3228, 2202, 1654, 1620, 1563, 1453, 1376, 1240, and 1031. ^1^H-NMR (300 MHz, DMSO-*d*_6_): *δ* 0.98 (s, 3H, CH_3_), 1.06 (s, 3H, CH_3_), 2.08 (d, ^2^*J* = 15.9 Hz, 1H, CH_2_), 2.26 (d, ^2^*J* = 15.9 Hz, 1H, CH_2_), 2.42 (d, ^2^*J* = 15.9 Hz, 1H, CH_2_), 2.58 (d, ^2^*J* = 15.9 Hz, 1H, CH_2_), 4.89 (s, 1H, CH), 6.35 (br s, 2H, NH_2_), 6.54 (br s, 2H, NH_2_), 7.07 (s, 1H, CH Ar), 7.63 (s, 1H, CH Ar), and 9.65 (br s, 1H, NH) ppm; ^13^C-NMR (75 MHz, DMSO-*d*_6_): *δ* 26.3, 27.5, 28.9, 31.7, 40.3, 50.3, 62.0, 98.2, 109.9, 110.3, 114.9, 116.8, 129.7, 130.3, 132.1, 146.6, 153.0, 154.6, 159.7, 164.4, and 196.2 ppm; MS (*m*/*z*, relative intensity %): 536 (^81^Br, ^81^Br, [M]^+^, 6), 534 (^81^Br, ^79^Br, [M]^+^, 14), 532 (^79^Br, ^79^Br, [M]^+^, 8), 452 (^81^Br, ^81^Br, 12), 450 (^81^Br, ^79^Br, 27), 448 (^79^Br, ^79^Br, 13), 385 (2), 371 (^81^Br, ^81^Br, 4), 369 (^81^Br, ^79^Br, 8), 367 (^79^Br, ^79^Br, 4), 283 (7), 227 (2), 220 (5), 150 (18), 83 (21), and 41 (100); Anal. calcd. for C_21_H_18_Br_2_N_4_O_3_: C, 47.22; H, 3.40; N, 10.49%; found: C, 47.16; H, 3.45; N, 10.44%.

***2,4-Diamino-5-(5,7-diiodo-3,3-dimethyl-1-oxo-2,3,4,9-tetrahydro-1H-xanthen-9-yl)-6-oxo-1,6-dihydropyridine-3-carbonitrile (2c):*** Yellowish solid; yield—98% (0.308 g); m.p. = 268–269 °C (decomp.) (from DMSO-H_2_O); FTIR (KBr) cm^−1^: 3317, 3206, 2199, 1617, 1578, 1475, 1440, 1374, 1243, and 1019. ^1^H-NMR (300 MHz, DMSO-*d*_6_): *δ* 0.98 (s, 3H, CH_3_), 1.06 (s, 3H, CH_3_), 2.07 (d, ^2^*J* = 16.2 Hz, 1H, CH_2_), 2.25 (d, ^2^*J* = 16.2 Hz, 1H, CH_2_), 2.42 (d, ^2^*J* = 16.2 Hz, 1H, CH_2_), 2.57 (d, ^2^*J* = 16.2 Hz, 1H, CH_2_), 4.83 (s, 1H, CH), 6.32 (br s, 2H, NH_2_), 6.53 (br s, 2H, NH_2_), 7.21 (s, 1H, CH Ar), 7.87 (s, 1H, CH Ar), and 9.61 (br s, 1H, NH) ppm; ^13^C-NMR (75 MHz, DMSO-*d*_6_): *δ* 26.3, 27.4, 28.9, 31.7, 40.3, 50.3, 86.1, 88.0, 98.3, 110.6, 116.8, 129.1, 136.5, 136.9, 143.1, 149.6, 152.9, 154.6, 159.6, 164.7, and 196.1 ppm; MS (*m*/*z*, relative intensity %): 628 ([M]^+^, 13), 544 (9), 463 (23), 336 (4), 283 (10), 209 (51), 150 (63), 127 (70), 41 (100), and 15 (25); Anal. calcd. for C_21_H_18_I_2_N_4_O_3_: C, 40.15; H, 2.89; N, 8.92%; found: C, 40.10; H, 2.96; N, 8.87%.

### 3.3. Thermal Rearrangement of 7,9-Dihalogenated 5-(2-Hydroxy-6-oxocyclohexyl)-5H-chromeno[2,3-b]pyridine-3-carbonitriles **1** at 150 °C

A solution of 7,9-dihalogenated 5-(2-hydroxy-6-oxocyclohexyl)-5*H*-chromeno[2,3-*b*]-pyridine-3-carbonitrile **1** (0.5 mmol) was stirred in DMSO (0.5 mL) for 1 h at 150 °C. Upon completion of the reaction, the reaction mixture was allowed to cool to room temperature, water (5 mL) was added to the reaction mixture, and the resulting precipitate of pure 3,5-dihalogenated 10-(2-hydroxyphenyl)-1-hydroxy-7,7-dimethyl-9-oxo-5,6,7,8,9,10-hexahydrobenzo[*b*][1,6]naphthyridine-4-carbonitrile **3** was separated by filtration, washed with cold ethanol (2 × 3 mL), and dried.

***3-Amino-10-(3,5-dichloro-2-hydroxyphenyl)-1-hydroxy-7,7-dimethyl-9-oxo-5,6,7,8,9,10-hexahydrobenzo[b][1,6]naphthyridine-4-carbonitrile (3a):*** Yellowish solid; yield—98% (0.218 g); m.p. = 274–275 °C (decomp.) (from DMSO-H_2_O); FTIR (KBr) cm^−1^: 3294, 3179, 2204, 1655, 1626, 1518, 1468, 1377, 1227, and 1010. ^1^H-NMR (300 MHz, DMSO-*d*_6_): *δ* 1.02 (s, 3H, CH_3_), 1.07 (s, 3H, CH_3_), 2.10 (d, ^2^*J* = 16.1 Hz, 1H, CH_2_), 2.28 (d, ^2^*J* = 16.1 Hz, 1H, CH_2_), 2.60 (d, ^2^*J* = 16.1 Hz, 1H, CH_2_), 2.79 (d, ^2^*J* = 16.1 Hz, 1H, CH_2_), 4.97 (s, 1H, CH), 6.72 (s, 1H, CH Ar), 7.03 (br s, 2H, NH_2_), 7.27 (s, 1H, CH Ar), 9.22 (br s, 1H, NH), and 11.25 (br s, 2H, 2 OH) ppm; ^13^C-NMR (75 MHz, DMSO-*d*_6_): *δ* 27.0, 28.2, 29.7, 32.5, 40.2, 50.2, 63.4, 96.5, 110.2, 115.2, 123.5, 123.8, 126.6, 127.3, 138.3, 146.4, 149.7, 153.0, 155.1, 162.6, and 195.4 ppm; MS (*m*/*z*, relative intensity %): 446 (^37^Cl, ^35^Cl, [M]^+^, 1), 444 (^35^Cl, ^35^Cl, [M]^+^, 1), 341 (1), 283 (^37^Cl, ^37^Cl, 1), 281 (^37^Cl, ^35^Cl, 3), 279 (^35^Cl, ^35^Cl, 4), 239 (1), 199 (1), 166 (^37^Cl, ^37^Cl, 2), 164(^37^Cl, ^35^Cl, 8), 162 (^35^Cl, ^35^Cl, 14), 113 (4), 78 (84), 63 (100), and 15 (54); Anal. calcd. for C_21_H_18_Cl_2_N_4_O_3_: C, 56.64; H, 4.07; N, 12.58%; found: C, 56.55; H, 4.12; N, 12.55%.

***3-Amino-10-(3,5-dibromo-2-hydroxyphenyl)-1-hydroxy-7,7-dimethyl-9-oxo-5,6,7,8,9,10-hexahydrobenzo[b][1,6]naphthyridine-4-carbonitrile (3b):*** Yellowish solid; yield—98% (0.262 g); m.p. = 267–268 °C (decomp.) (from DMSO-H_2_O); FTIR (KBr) cm^−1^: 3289, 3180, 2204, 1655, 1626, 1518, 1466, 1376, 1226, and 1010. ^1^H-NMR (300 MHz, DMSO-*d*_6_): *δ* 1.03 (s, 3H, CH_3_), 1.08 (s, 3H, CH_3_), 2.09 (d, ^2^*J* = 16.1 Hz, 1H, CH_2_), 2.29 (d, ^2^*J* = 16.1 Hz, 1H, CH_2_), 2.60 (d, ^2^*J* = 16.1 Hz, 1H, CH_2_), 2.79 (d, ^2^*J* = 16.1 Hz, 1H, CH_2_), 4.96 (s, 1H, CH), 6.88 (s, 1H, CH Ar), 7.04 (br s, 2H, NH_2_), 7.50 (s, 1H, CH Ar), 9.23 (s, 1H, NH), 11.25 (br s, 1H, OH), and 11.39 (s, 1H, OH) ppm; ^13^C-NMR (75 MHz, DMSO-*d*_6_): *δ* 26.4, 27.8, 29.3, 32.0, 39.7, 49.7, 63.0, 96.0, 109.8, 111.0, 113.2, 114.6, 129.6, 132.2, 137.9, 146.0, 150.5, 152.5, 154.5, 162.2, and 194.8 ppm; MS (*m*/*z*, relative intensity %): 536 (^81^Br, ^81^Br, [M]^+^, 6), 534 (^81^Br, ^79^Br, [M]^+^, 11), 532 (^79^Br, ^79^Br, [M]^+^, 7), 437 (^81^Br, ^81^Br, 1), 435 (^81^Br, ^79^Br, 2), 433 (^79^Br, ^79^Br, 1), 384 (3), 371 (^81^Br, ^81^Br, 4), 369 (^81^Br, ^79^Br, 8), 367 (^79^Br, ^79^Br, 6), 283 (61), 254 (^81^Br, ^81^Br, 33), 252 (^81^Br, ^79^Br, 74), 250 (^79^Br, ^79^Br, 36), 172 (6), 143 (9), 78 (61), and 15 (100); Anal. calcd. for C_21_H_18_Br_2_N_4_O_3_: C, 47.22; H, 3.40; N, 10.49%; found: C, 47.16; H, 3.49; N, 10.44%.

***3-Amino-1-hydroxy-10-(2-hydroxy-3,5-diiodophenyl)-7,7-dimethyl-9-oxo-5,6,7,8,9,10-hexahydrobenzo[b][1,6]naphthyridine-4-carbonitrile (3c)***: Yellowish solid; yield—90% (0.283 g); m.p. = 258–259 °C (decomp.) (from DMSO-H_2_O); FTIR (KBr) cm^−1^: 3412, 3192, 2204, 1627, 1515, 1460, 1373, 1295, 1225, and 1013. ^1^H-NMR (300 MHz, DMSO-*d*_6_): *δ* 1.02 (s, 3H, CH_3_), 1.06 (s, 3H, CH_3_), 2.05 (d, ^2^*J* = 16.0 Hz, 1H, CH_2_), 2.28 (d, ^2^*J* = 16.0 Hz, 1H, CH_2_), 2.59 (d, ^2^*J* = 16.0 Hz, 1H, CH_2_), 2.76 (d, ^2^*J* = 16.0 Hz, 1H, CH_2_), 4.89 (s, 1H, CH), 6.97–7.09 (m, 3H, NH_2_ + CH Ar), 7.73 (s, 1H, CH Ar), 9.20 (s, 1H, NH), 11.22 (br s, 1H, OH), and 11.48 (s, 1H, OH) ppm; ^13^C-NMR (75 MHz, DMSO-*d*_6_): *δ* 26.7, 28.3, 30.0, 32.4, 50.1, 63.5, 83.5, 90.5, 96.7, 110.4, 115.0, 136.9, 137.4, 141.5, 143.8, 146.4, 153.0, 153.9, 154.9, 162.8, and 195.6 ppm; MS (*m*/*z*, relative intensity %): 628 ([M]^+^, 1), 592 (6), 467 (1), 346 (100), 283 (7), 220 (10), 191 (4), 127 (14), 92 (7), and 18 (12); Anal. calcd. for C_21_H_18_I_2_N_4_O_3_: C, 40.15; H, 2.89; N, 8.92%; found: C, 40.10; H, 2.96; N, 8.88%.

### 3.4. Thermal Rearrangement of 5,7-Dihalogenated 5-(1-oxo-2,3,4,9-tetrahydro-1H-xanthen-9-yl)-6-oxo-1,6-dihydropyridine-3-carbonitriles **2** at 150 °C

A solution of 5,7-dihalogenated 5-(1-oxo-2,3,4,9-tetrahydro-1*H*-xanthen-9-yl)-6-oxo-1,6-dihydropyridine-3-carbonitriles **2** (0.5 mmol) was stirred in DMSO (0.5 mL) for 1 h at 150 °C. Upon completion of the reaction, the reaction mixture was allowed to cool to room temperature, water (5 mL) was added to the reaction mixture, and the resulting precipitate of pure 3,5-dihalogenated 10-(2-hydroxyphenyl)-1-hydroxy-7,7-dimethyl-9-oxo-5,6,7,8,9,10-hexahydrobenzo[*b*][1,6]naphthyridine-4-carbonitrile **3** was separated by filtration, washed with cold ethanol (2 × 3 mL), and dried.

***3-Amino-10-(3,5-dichloro-2-hydroxyphenyl)-1-hydroxy-7,7-dimethyl-9-oxo-5,6,7,8,9,10-hexahydrobenzo[b][1,6]naphthyridine-4-carbonitrile (3a):*** Yellowish solid; yield—97% (0.216 g); m.p. = 274–275 °C (decomp.) (from DMSO-H_2_O); ^1^H-NMR (300 MHz, DMSO-*d*_6_): *δ* 1.02 (s, 3H, CH_3_), 1.07 (s, 3H, CH_3_), 2.10 (d, ^2^*J* = 16.1 Hz, 1H, CH_2_), 2.28 (d, ^2^*J* = 16.1 Hz, 1H, CH_2_), 2.61 (d, ^2^*J* = 16.1 Hz, 1H, CH_2_), 2.79 (d, ^2^*J* = 16.1 Hz, 1H, CH_2_), 4.97 (s, 1H, CH), 6.72 (s, 1H, CH Ar), 7.04 (br s, 2H, NH_2_), 7.27 (s, 1H, CH Ar), 9.23 (br s, 1H, NH), and 11.24 (br s, 2H, 2 OH) ppm.

***3-Amino-10-(3,5-dibromo-2-hydroxyphenyl)-1-hydroxy-7,7-dimethyl-9-oxo-5,6,7,8,9,10-hexahydrobenzo[b][1,6]naphthyridine-4-carbonitrile (3b):*** Yellowish solid; yield—97% (0.259 g); m.p. = 267–268 °C (decomp.) (from DMSO-H_2_O); ^1^H-NMR (300 MHz, DMSO-*d*_6_): *δ* 1.03 (s, 3H, CH_3_), 1.08 (s, 3H, CH_3_), 2.09 (d, ^2^*J* = 16.1 Hz, 1H, CH_2_), 2.29 (d, ^2^*J* = 16.1 Hz, 1H, CH_2_), 2.61 (d, ^2^*J* = 16.1 Hz, 1H, CH_2_), 2.79 (d, ^2^*J* = 16.1 Hz, 1H, CH_2_), 4.96 (s, 1H, CH), 6.88 (s, 1H, CH Ar), 7.05 (br s, 2H, NH_2_), 7.50 (s, 1H, CH Ar), 9.23 (s, 1H, NH), 11.26 (br s, 1H, OH), and 11.39 (s, 1H, OH) ppm.

***3-Amino-1-hydroxy-10-(2-hydroxy-3,5-diiodophenyl)-7,7-dimethyl-9-oxo-5,6,7,8,9,10-hexahydrobenzo[b][1,6]naphthyridine-4-carbonitrile (3c)***: Yellowish solid; yield—93% (0.292 g); m.p. = 258–259 °C (decomp.) (from DMSO-H_2_O); ^1^H-NMR (300 MHz, DMSO-*d*_6_): *δ* 1.02 (s, 3H, CH_3_), 1.06 (s, 3H, CH_3_), 2.05 (d, ^2^*J* = 16.0 Hz, 1H, CH_2_), 2.27 (d, ^2^*J* = 16.0 Hz, 1H, CH_2_), 2.59 (d, ^2^*J* = 16.0 Hz, 1H, CH_2_), 2.76 (d, ^2^*J* = 16.0 Hz, 1H, CH_2_), 4.89 (s, 1H, CH), 6.97–7.10 (m, 3H, NH_2_ + CH Ar), 7.73 (s, 1H, CH Ar), 9.21 (s, 1H, NH), 11.22 (br s, 1H, OH), and 11.48 (s, 1H, OH) ppm.

## 4. Conclusions

In summary, the thermal rearrangement of 7,9-dihalogen-substituted 5-(2-hydroxy-6-oxocyclohexyl)-5*H*-chromeno[2,3-*b*]pyridine-3-carbonitriles into previously unknown 5,7-dihalogenated 5-(2,3,4,9-tetrahydro-1*H*-xanthen-9-yl)-6-oxo-1,6-di-hydropyridines and 10-(3,5-dihalogen-2-hydroxyphenyl)-5,6,7,8,9,10-hexahydrobenzo-[*b*][1,6]naphthyridines was observed. The developed approach is facile and easy for isolating pure final compounds directly from the reaction mixture using the addition of water, and the yields of the final compounds are 90–99%.

The proposed structures of synthesized 2,3,4,9-tetrahydro-1*H*-xanthenes and 5,6,7,8,9,10-hexahydrobenzo[*b*][1,6]naphthyridines were clearly confirmed by 2D NMR spectroscopy.

During the investigation of the reaction mechanism using ^1^H-NMR monitoring at temperatures of 100 °C and 150 °C, it was found that the reaction proceeded in polar solvents. The reaction occurred via the ANRORC mechanism. It was found that the reaction occurred rapidly. Heating at 100 °C resulted in 2,3,4,9-tetrahydro-1*H*-xanthene formation; heating at 150 °C also lead to 2,3,4,9-tetrahydro-1*H*-xanthene, which then transformed into 5,6,7,8,9,10-hexahydrobenzo[*b*][1,6]naphthyridine.

## Figures and Tables

**Table 1:** Optimization of thermal rearrangement conditions ^1^.

Entry	Solvent, mL	Temperature, °C	Time, h	Yield of 2a, %	Yield of 3a, %
1	DMSO, 0.5	100	1	99 ^2^	–
2	DMSO, 0.5	120	1	88	10
3	DMSO, 0.5	150	1	–	98 ^2^
4	DMF, 0.5	100	1	95 ^2^	–
5	DMF, 0.5	150	1	–	94 ^2^
6	H_2_O, 2	100	1	6	5
7	MeCN, 0.5	82	1	8	–
8	*n*-PrOH, 0.5	97	1	25	–
9	Dioxane, 0.5	101	1	–	–
10	DMSO, 0.5	100	0.5	82 ^2^	–
11	DMSO, 0.5	150	0.5	–	80 ^2^

^1^ We heated 5-(2-hydroxy-6-oxocyclohexyl)-5*H*-chromeno[2,3-*b*]pyridine **1a** (0.5 mmol) in the corresponding solvent. ^2^ Yields of isolated compounds **2a** and **3a** (in other cases according to NMR data).

**Table 2:** Thermal rearrangement of 7,9-dihalogen-substituted 5-(2-hydroxy-6-oxocyclohexyl)-5*H*-chromeno[2,3-*b*]pyridines **1** ^1^.

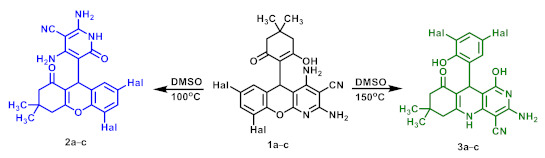
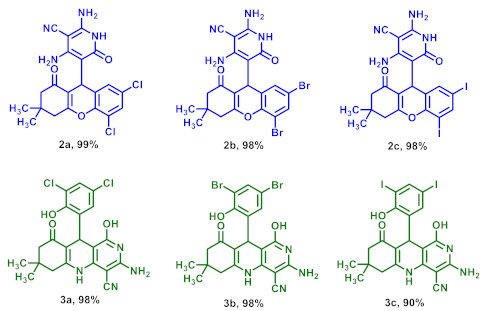

^1^ Isolated yields. Procedure for compounds **2**: a solution of 0.5 mmol 7,9-dihalogenated chromeno[2,3-*b*]pyridine-3-carbonitrile **1** was stirred in 0.5 mL DMSO for 1 h at 100 °C. Procedure for compounds **3**: a solution of 0.5 mmol of 7,9-dihalogenated chromeno[2,3-*b*]pyridine-3-carbonitrile **1** was stirred in 0.5 mL of DMSO for 1 h at 150 °C.

**Table 3:** Verification of the generality of the thermal rearrangement of chromeno[2,3-*b*]pyridines **1** with other substituents ^1,2^.

Entry	Chromeno[2,3-*b*]pyridine 1	Heating in DMSO at 100 °C		Heating in DMSO at 150 °C	
		Yield of 2, %	Yield of 3, %	Yield of 2, %	Yield of 3, %
1	**1d**	–	–	Decomposition	
2	**1e**	–	–	Decomposition	
3	**1f**	7	–	3	7
4	**1g**	11	–	4	7
5	**1h**	–	–	Decomposition	
6	**1j**	80	–	–	78
7	**1k**	82	–	–	80

^1^ We heated 5-(2-hydroxy-6-oxocyclohexyl)-5*H*-chromeno[2,3-*b*]pyridine **1** (0.5 mmol) in DMSO (0.5 mL) at 100 °C or 150 °C within 1 h. ^2^ Yields according to NMR data.

## Data Availability

Data are contained within the article or Supplementary Materials.

## Supplementary Materials

The following supporting information can be downloaded at: https://www.mdpi.com/article/10.3390/molecules28073139/s1, Figures S1–S6: The ^1^H and ^13^C spectra of synthesized compounds **2a–c**; Figures S7–S12: The ^1^H and ^13^C spectra of synthesized compounds **3a–c**; Figures S13–S16: The ^15^N- and 2D-NMR spectra of **2a**; Figures S17–S20: The ^15^N- and 2D-NMR spectra of **3a**.

## Sample Availability

Samples of the compounds **1**, **2,** and **3** are available from the authors.
