# Supplementary material for: Thermal Rearrangement of 5-(2-Hydroxy-6-oxocyclohexyl)-5H-chromeno[2,3-b]pyridines
Source: Molecules. 2023 Mar 31;28(7):3139. doi: 10.3390/molecules28073139 (PMC10095857; doi:10.3390/molecules28073139)
Supplement: Supplementary file 1 [file molecules-28-03139-s001.zip › molecules-2310609-supplementary.pdf]

Supplementary Material:

Table of Contents

1. The copies of ^{1}H NMR and ^{13}C NMR spectra for compounds **2a-c**....................................... S2

2. The copies of ^{1}H NMR and ^{13}C NMR spectra for compounds **3a-c**....................................... S5

3. ^{15}N and 2D NMR spectra for compound **2a**.. S8

4. ^{15}N and 2D NMR spectra for compound **3a**.. S10

1. The copies of ^{1}H NMR and ^{13}C NMR spectra for compounds 2a-c

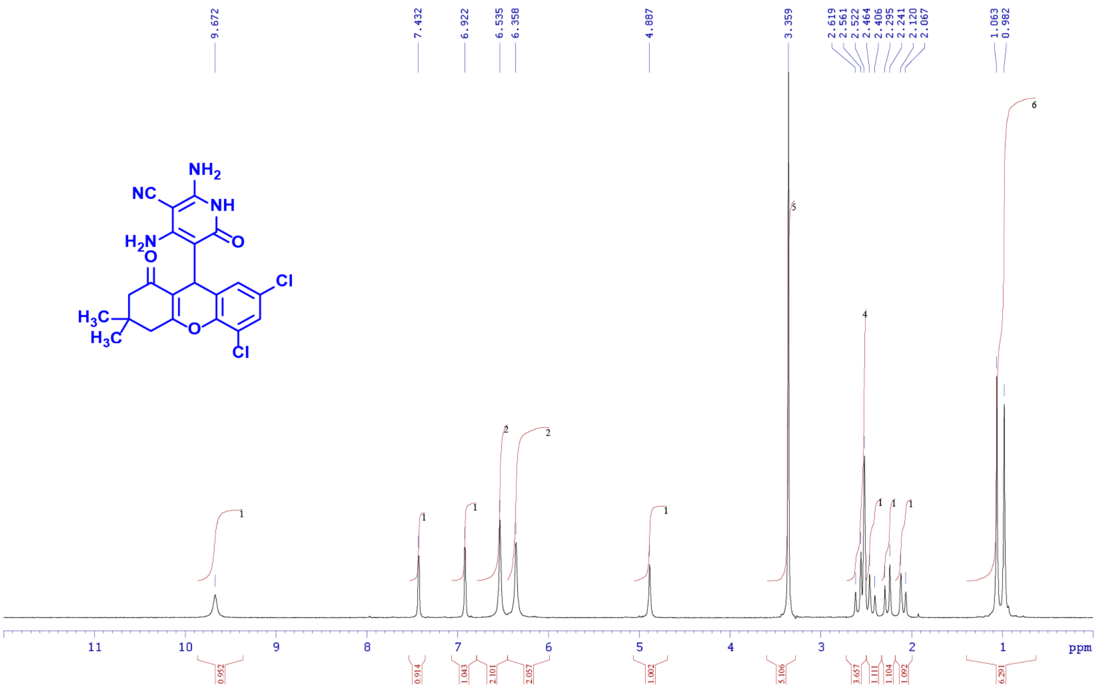

Figure S1. ^{1}H NMR spectrum of 2,4-diamino-5-(5,7-dichloro-3,3-dimethyl-1-oxo-2,3,4,9-tetrahydro-1*H*-xanthen-9-yl)-6-oxo-1,6-dihydropyridine-3-carbonitrile **2a** in DMSO-*d*6.

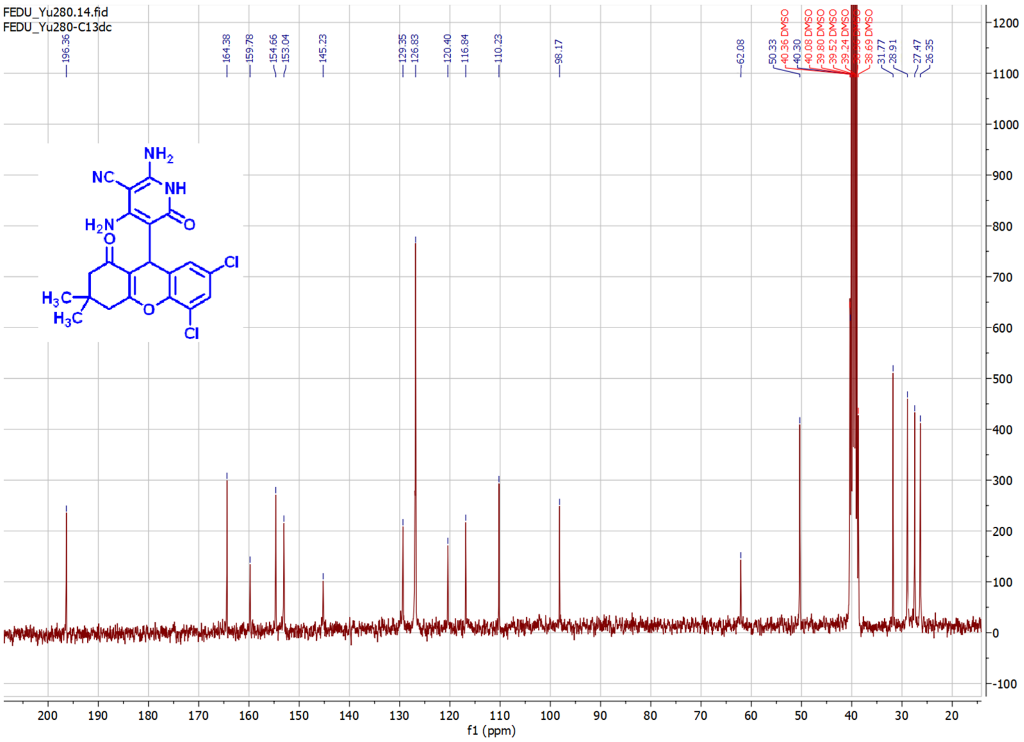

Figure S2. ^{13}C NMR spectrum of 2,4-diamino-5-(5,7-dichloro-3,3-dimethyl-1-oxo-2,3,4,9-tetrahydro-1*H*-xanthen-9-yl)-6-oxo-1,6-dihydropyridine-3-carbonitrile **2a** in DMSO-*d*6.

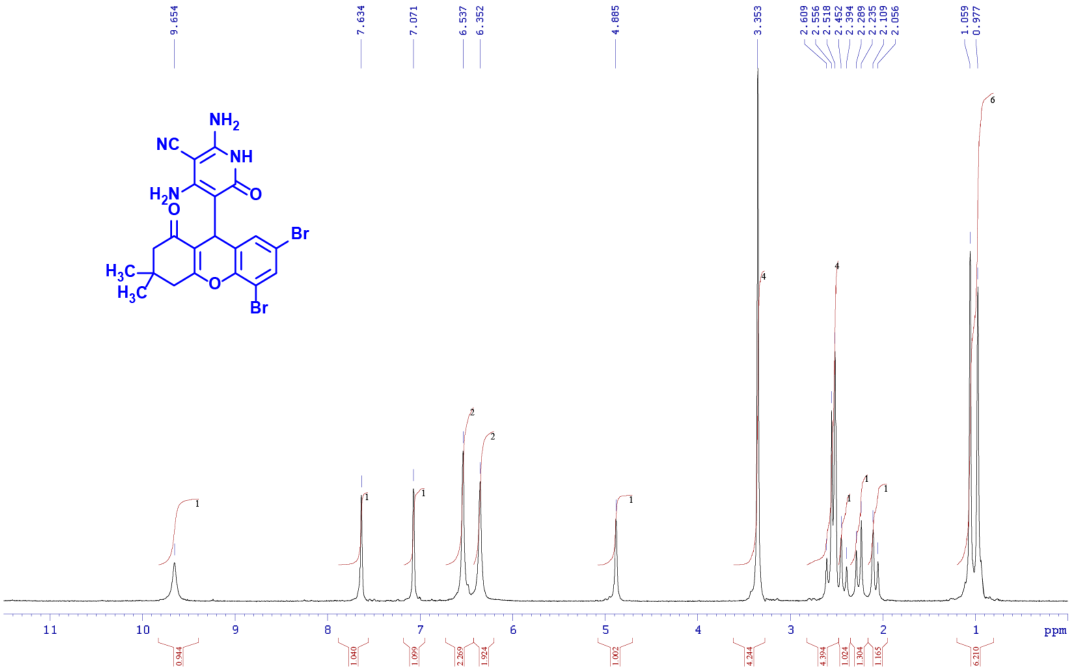

Figure S3. ^{1}H NMR spectrum of 2,4-diamino-5-(5,7-dibromo-3,3-dimethyl-1-oxo-2,3,4,9-tetrahydro-1*H*-xanthen-9-yl)-6-oxo-1,6-dihydropyridine-3-carbonitrile **2b** in DMSO-*d*6.

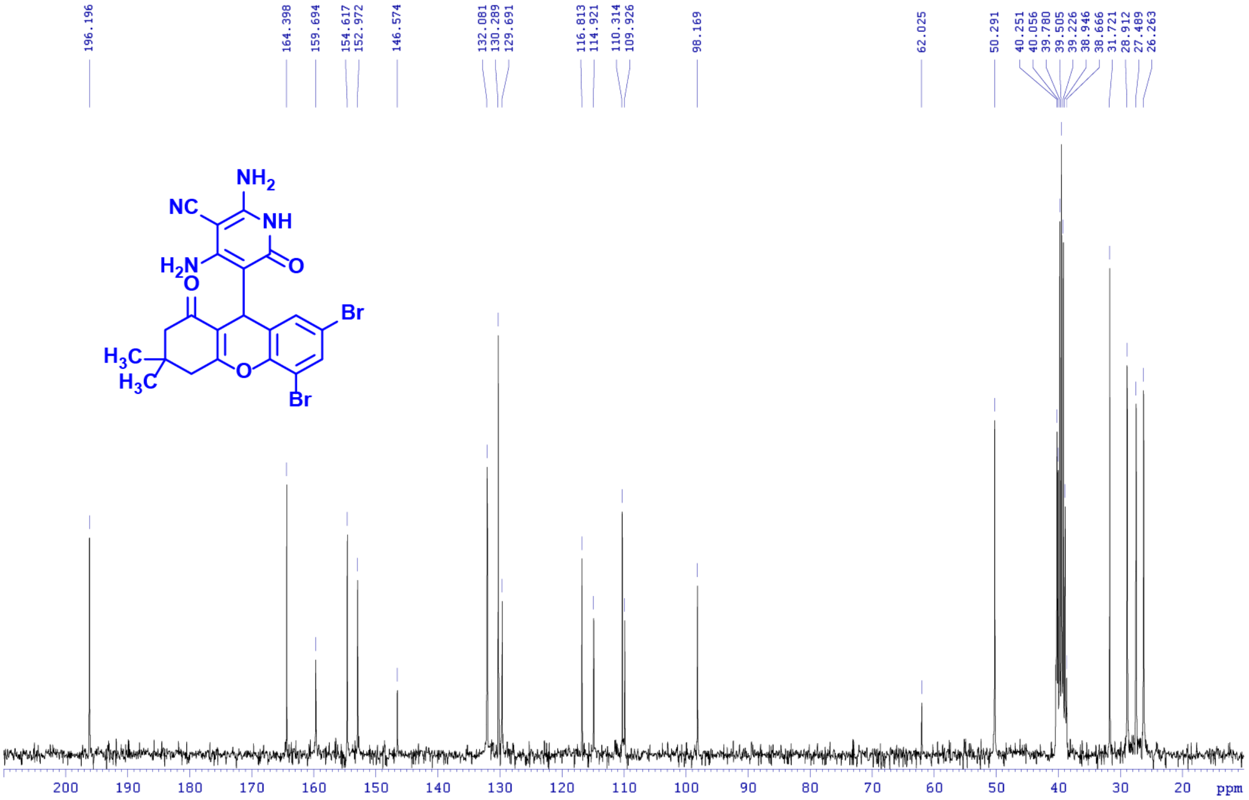

Figure S4. ^{13}C NMR spectrum of 2,4-diamino-5-(5,7-dibromo-3,3-dimethyl-1-oxo-2,3,4,9-tetrahydro-1*H*-xanthen-9-yl)-6-oxo-1,6-dihydropyridine-3-carbonitrile **2b** in DMSO-*d*6.

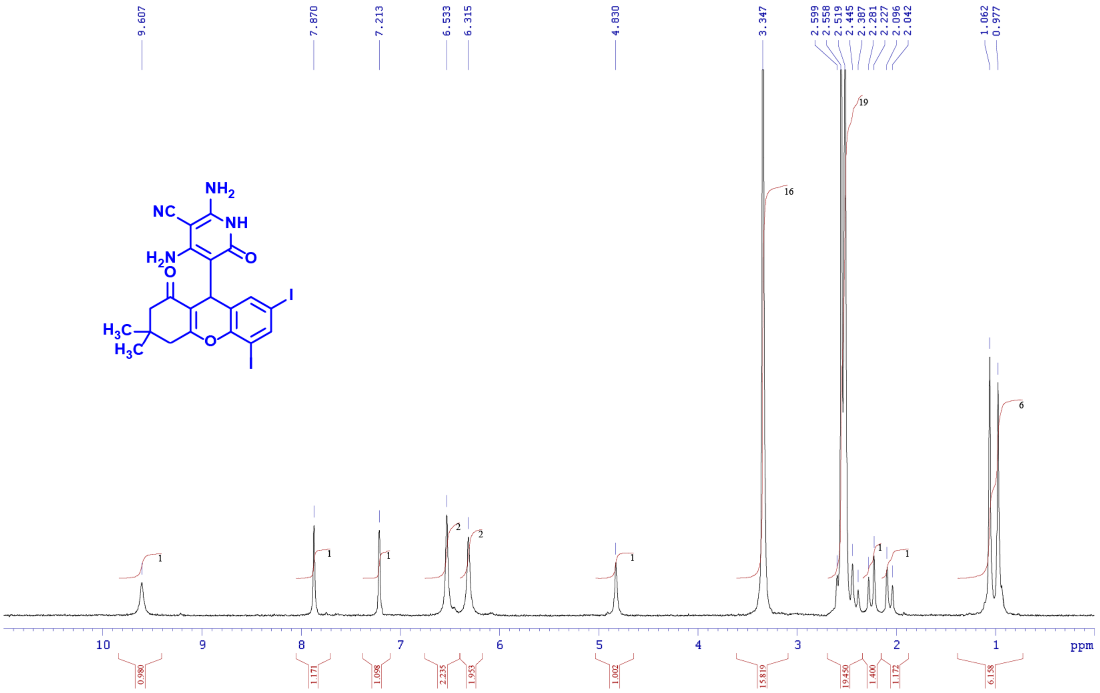

Figure S5. ^{1}H NMR spectrum of 2,4-diamino-5-(5,7-diiodo-3,3-dimethyl-1-oxo-2,3,4,9-tetrahydro-1*H*-xanthen-9-yl)-6-oxo-1,6-dihydropyridine-3-carbonitrile **2c** in DMSO-*d*6.

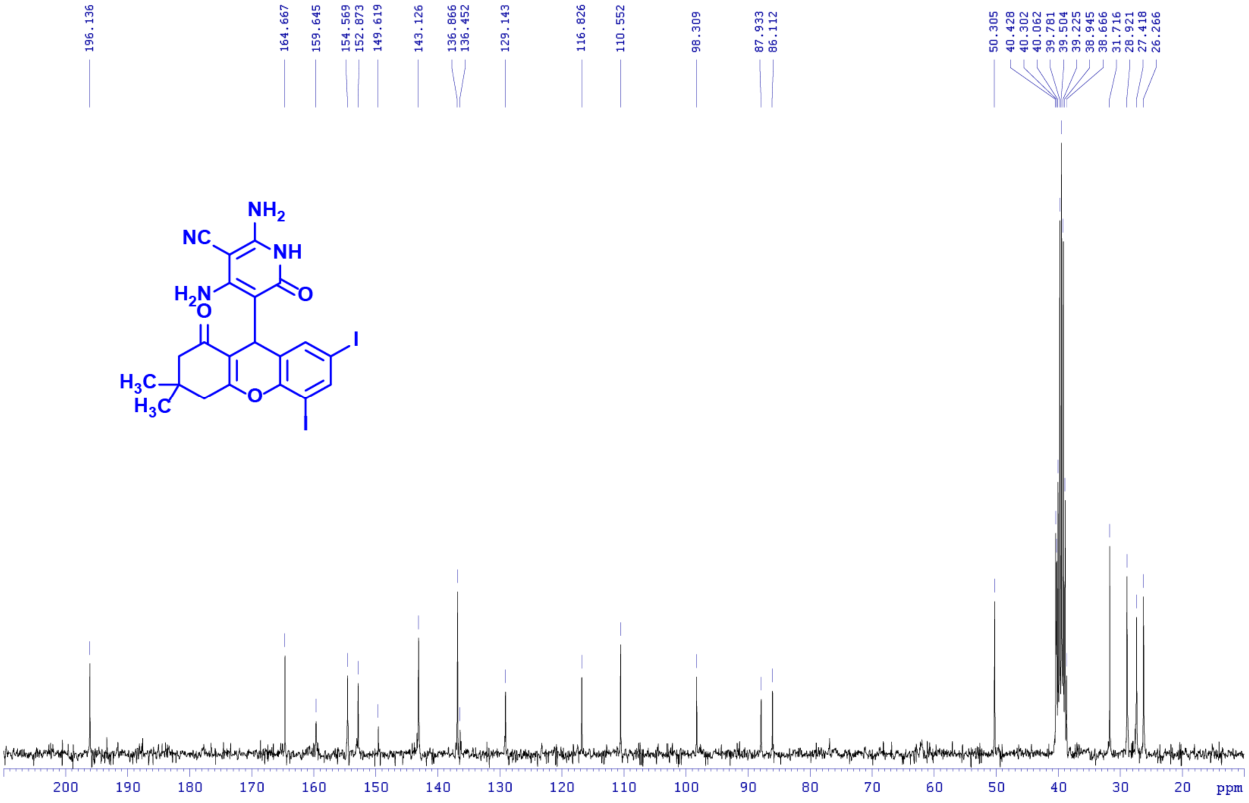

Figure S6. ^{13}C NMR spectrum of 2,4-diamino-5-(5,7-diiodo-3,3-dimethyl-1-oxo-2,3,4,9-tetrahydro-1*H*-xanthen-9-yl)-6-oxo-1,6-dihydropyridine-3-carbonitrile **2c** in DMSO-*d*6.

2. The copies of ^{1}H NMR and ^{13}C NMR spectra for compounds 3a-c

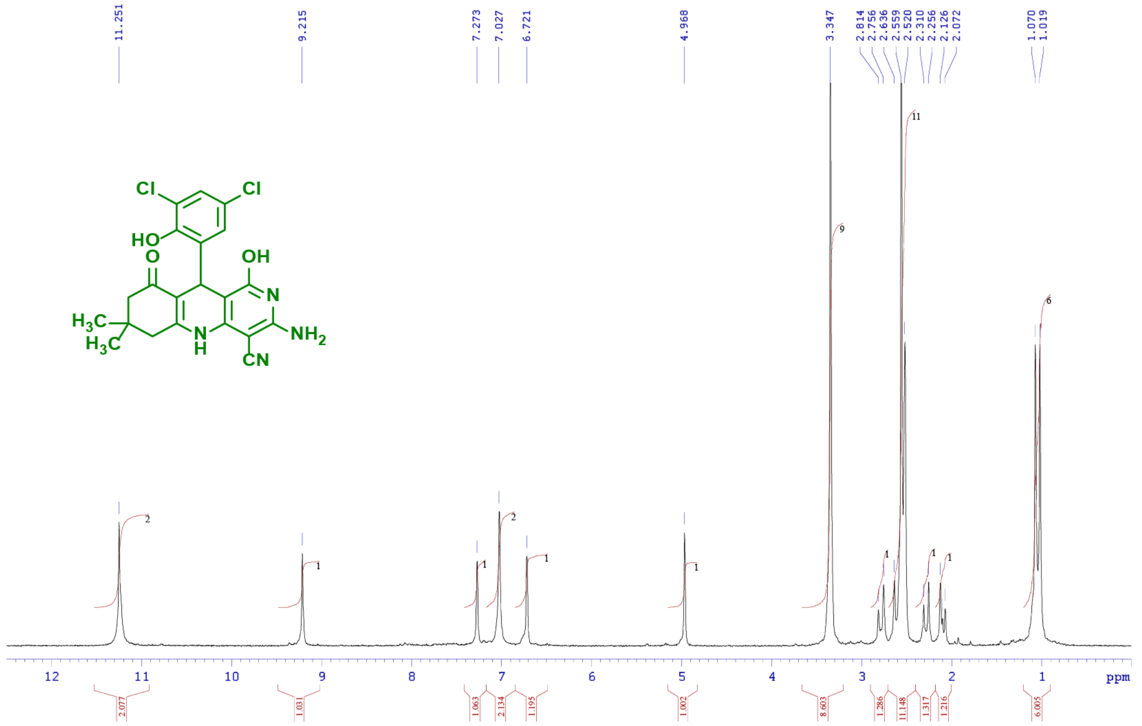

Figure S7. ^{1}H NMR spectrum of 3-amino-10-(3,5-dichloro-2-hydroxyphenyl)-1-hydroxy-7,7-dimethyl-9-oxo-5,6,7,8,9,10-hexahydrobenzo[*b*][1,6]naphthyridine-4-carbonitrile **3a** in DMSO-*d*6.

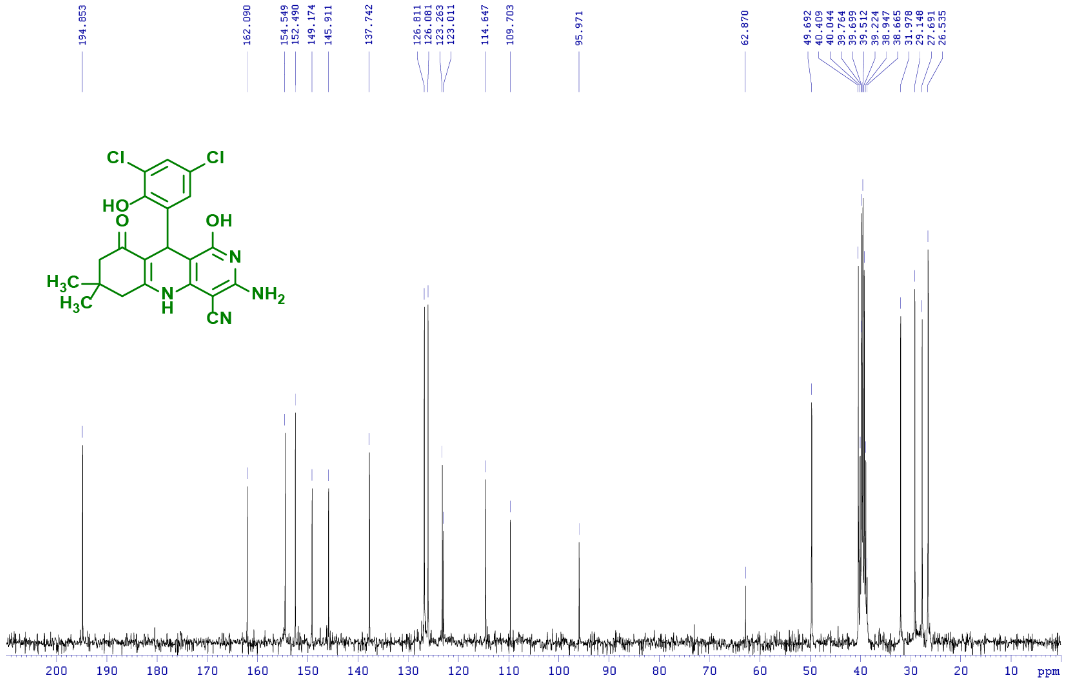

Figure S8. ^{13}C NMR spectrum of 3-amino-10-(3,5-dichloro-2-hydroxyphenyl)-1-hydroxy-7,7-dimethyl-9-oxo-5,6,7,8,9,10-hexahydrobenzo[*b*][1,6]naphthyridine-4-carbonitrile **3a** in DMSO-*d*6.

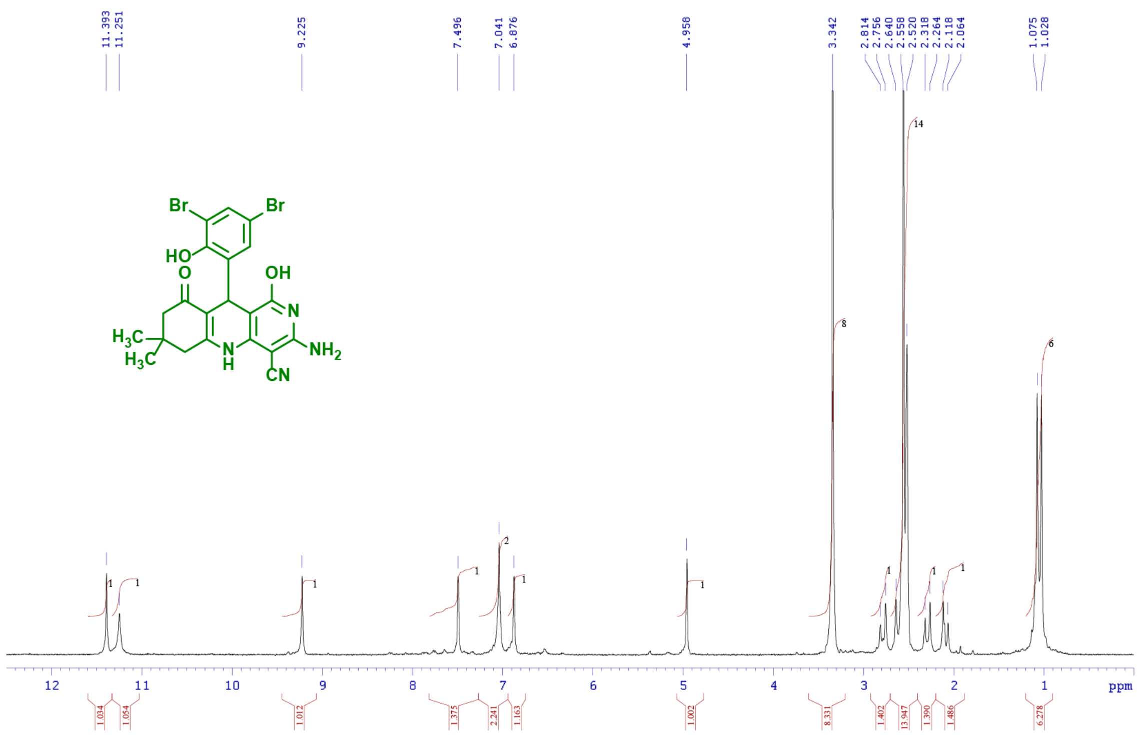

Figure S9. ^{1}H NMR spectrum of 3-amino-10-(3,5-dibromo-2-hydroxyphenyl)-1-hydroxy-7,7-dimethyl-9-oxo-5,6,7,8,9,10-hexahydrobenzo[*b*][1,6]naphthyridine-4-carbonitrile **3b** in DMSO-*d*6.

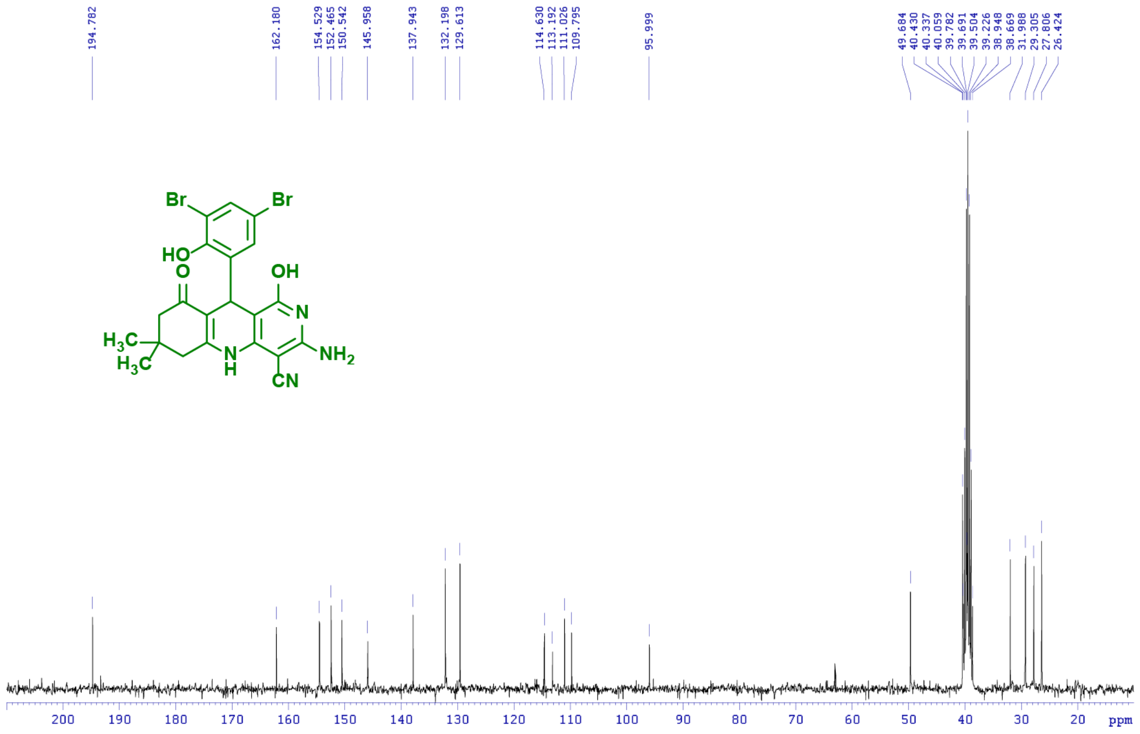

Figure S10. ^{13}C NMR spectrum of 3-amino-10-(3,5-dibromo-2-hydroxyphenyl)-1-hydroxy-7,7-dimethyl-9-oxo-5,6,7,8,9,10-hexahydrobenzo[*b*][1,6]naphthyridine-4-carbonitrile **3b** in DMSO-*d*6.

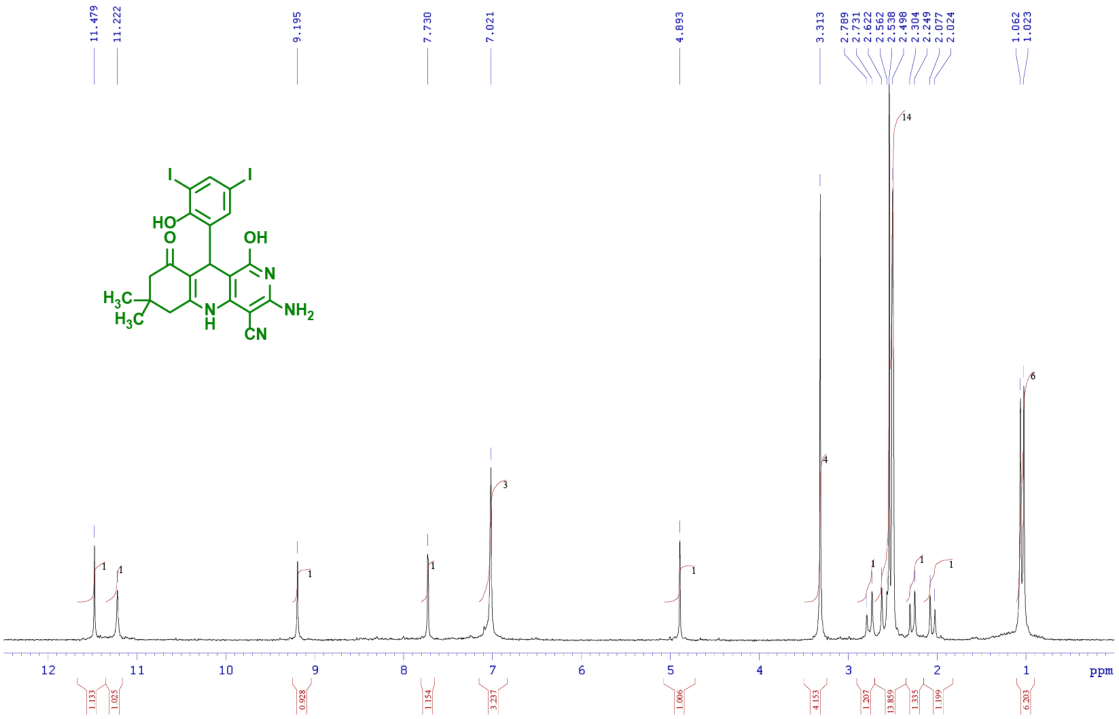

Figure S11. ^{1}H NMR spectrum of 3-amino-1-hydroxy-10-(2-hydroxy-3,5-diiodophenyl)-7,7-dimethyl-9-oxo-5,6,7,8,9,10-hexahydrobenzo[*b*][1,6]naphthyridine-4-carbonitrile **3c** in DMSO-*d*6.

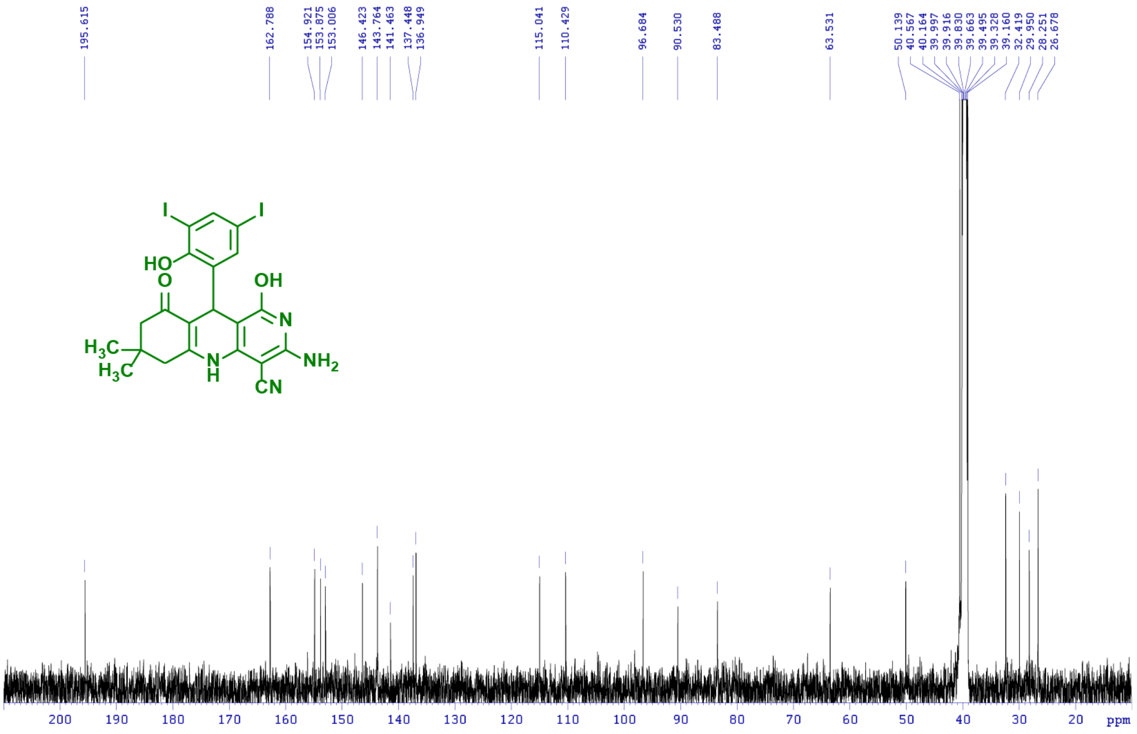

Figure S12. ^{13}C NMR spectrum of 3-amino-1-hydroxy-10-(2-hydroxy-3,5-diiodophenyl)-7,7-dimethyl-9-oxo-5,6,7,8,9,10-hexahydrobenzo[*b*][1,6]naphthyridine-4-carbonitrile **3c** in DMSO-*d*6.

3. ^{15}N and 2D NMR spectra for compound 2a

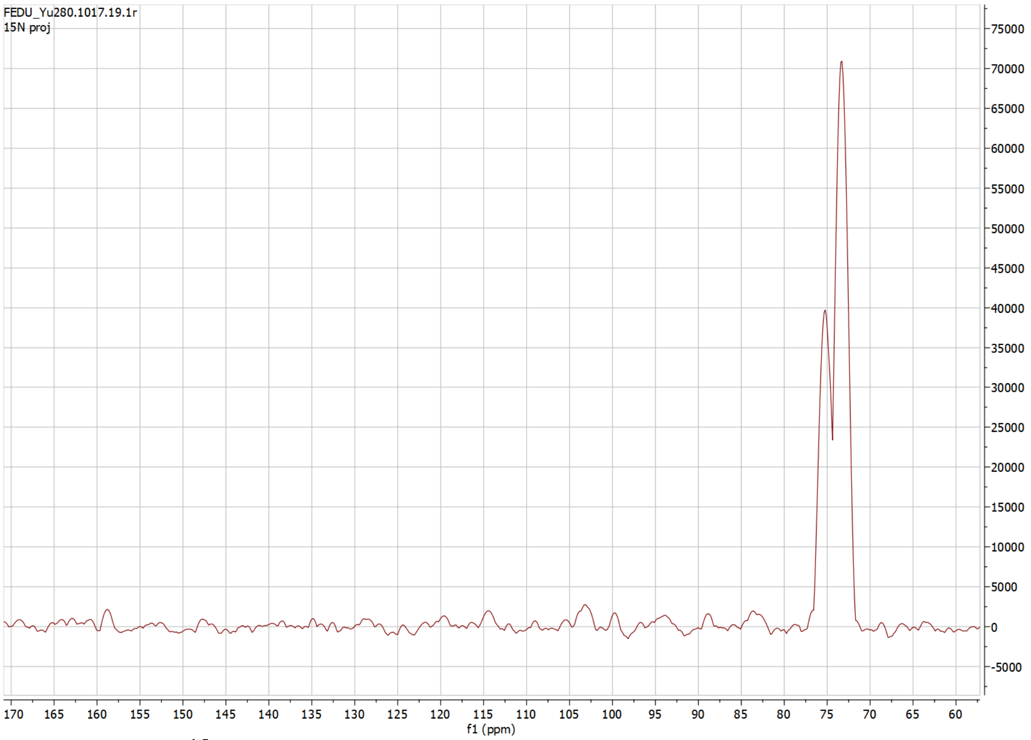

Figure S13. ^{15}N NMR spectrum of 2,4-diamino-5-(5,7-dichloro-3,3-dimethyl-1-oxo-2,3,4,9-tetrahydro-1*H*-xanthen-9-yl)-6-oxo-1,6-dihydropyridine-3-carbonitrile **2a** in DMSO-d_6.

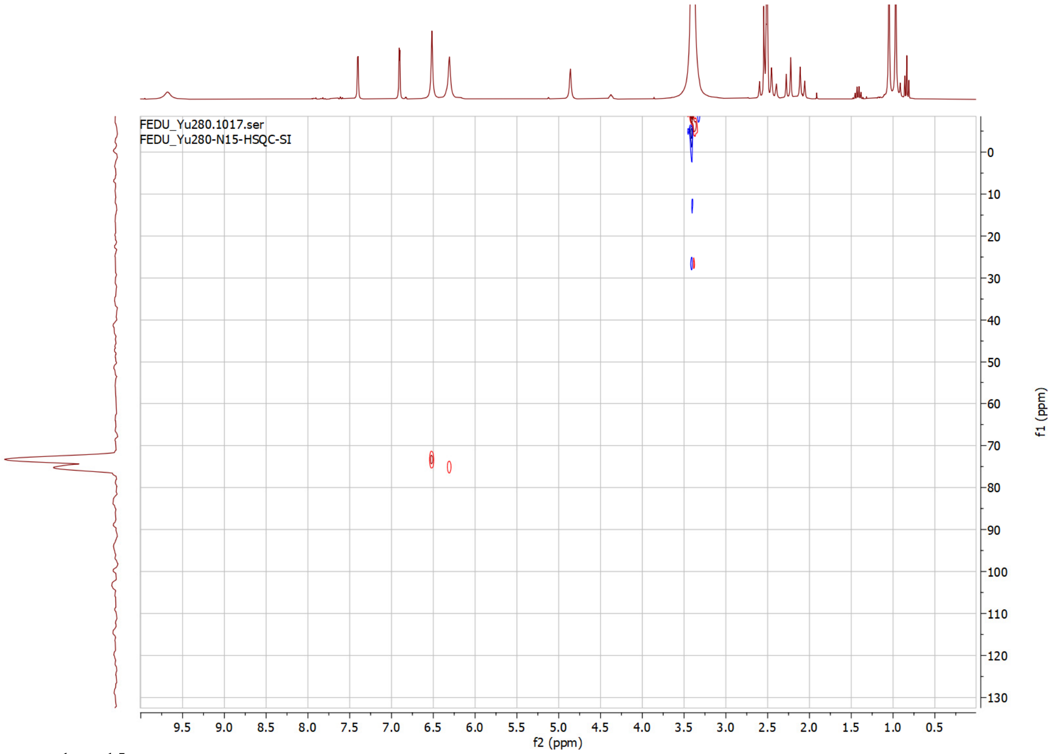

Figure S14. ^{1}H-^{15}N HSQC NMR spectrum of 2,4-diamino-5-(5,7-dichloro-3,3-dimethyl-1-oxo-2,3,4,9-tetrahydro-1*H*-xanthen-9-yl)-6-oxo-1,6-dihydropyridine-3-carbonitrile **2a** in DMSO-d_6.

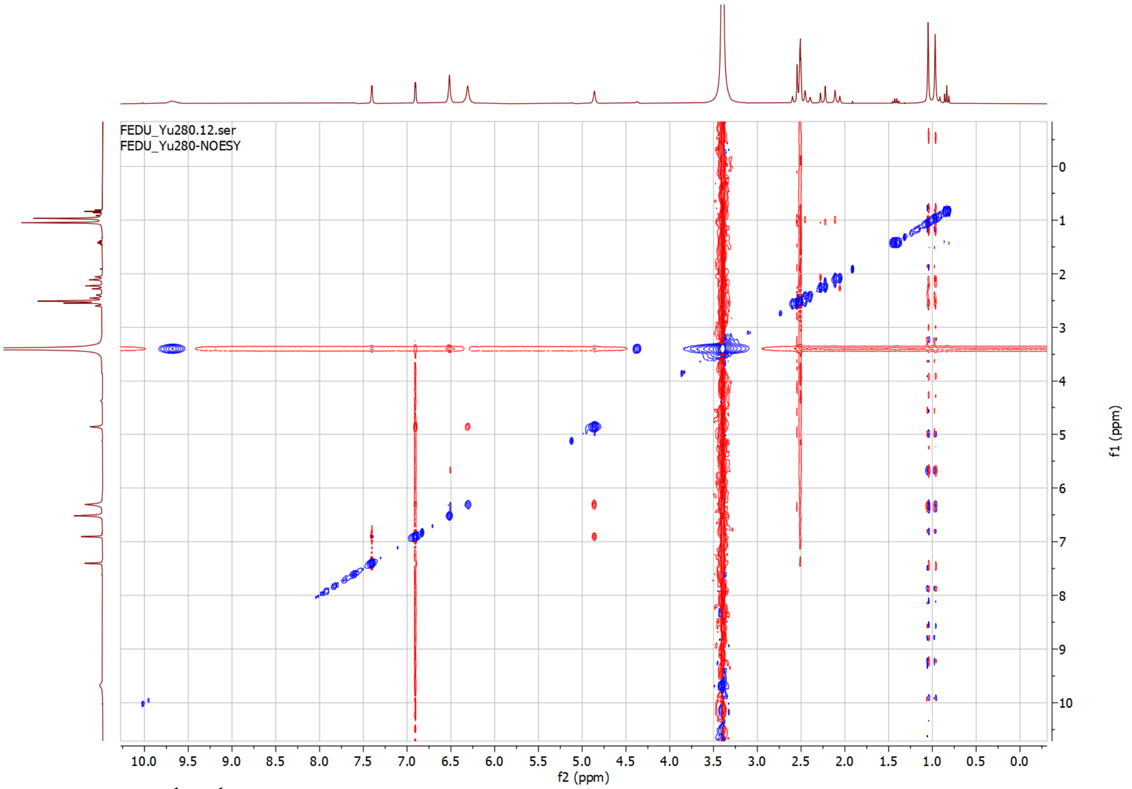

Figure S15. ^{1}H-^{1}H NOESY NMR spectrum of 2,4-diamino-5-(5,7-dichloro-3,3-dimethyl-1-oxo-2,3,4,9-tetrahydro-1*H*-xanthen-9-yl)-6-oxo-1,6-dihydropyridine-3-carbonitrile **2a** in DMSO-*d*$_6$.

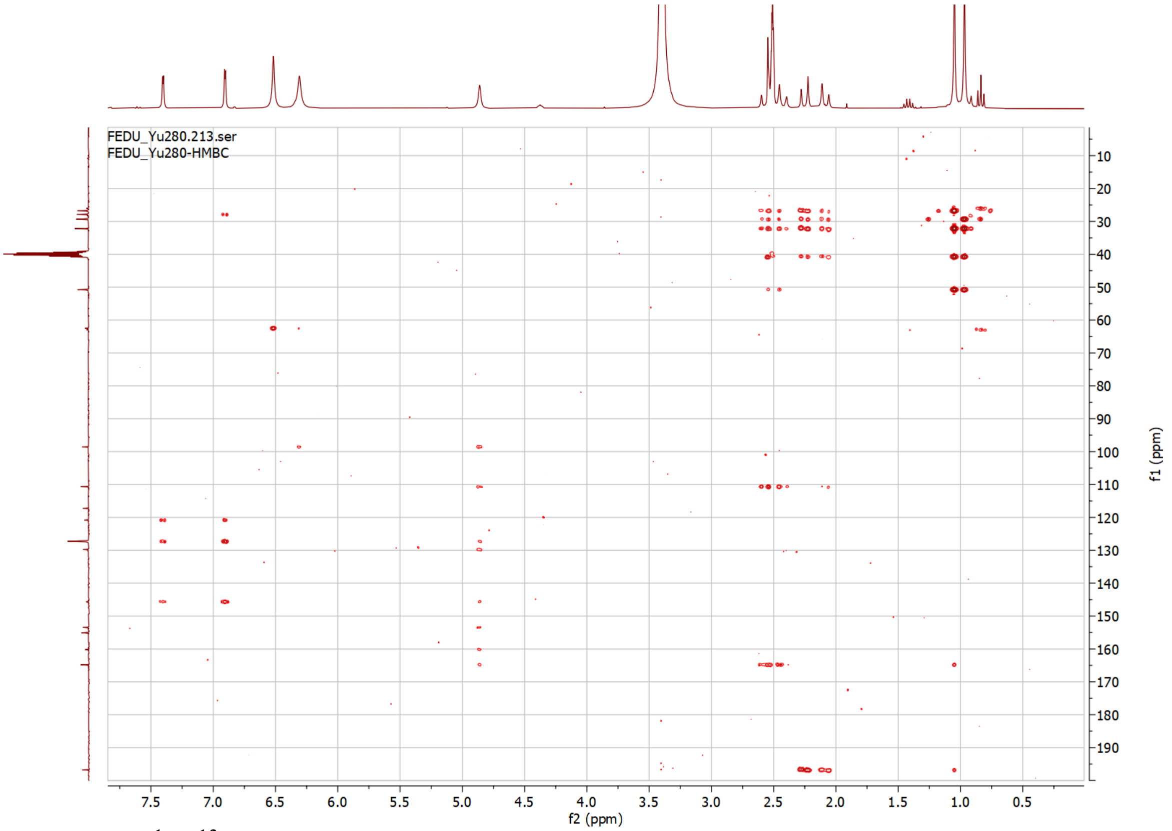

Figure S16. ^{1}H-^{13}C HMBC NMR spectrum of 2,4-diamino-5-(5,7-dichloro-3,3-dimethyl-1-oxo-2,3,4,9-tetrahydro-1*H*-xanthen-9-yl)-6-oxo-1,6-dihydropyridine-3-carbonitrile **2a** in DMSO-*d*$_6$.

4. ^{15}N and 2D NMR spectra for compound 3a

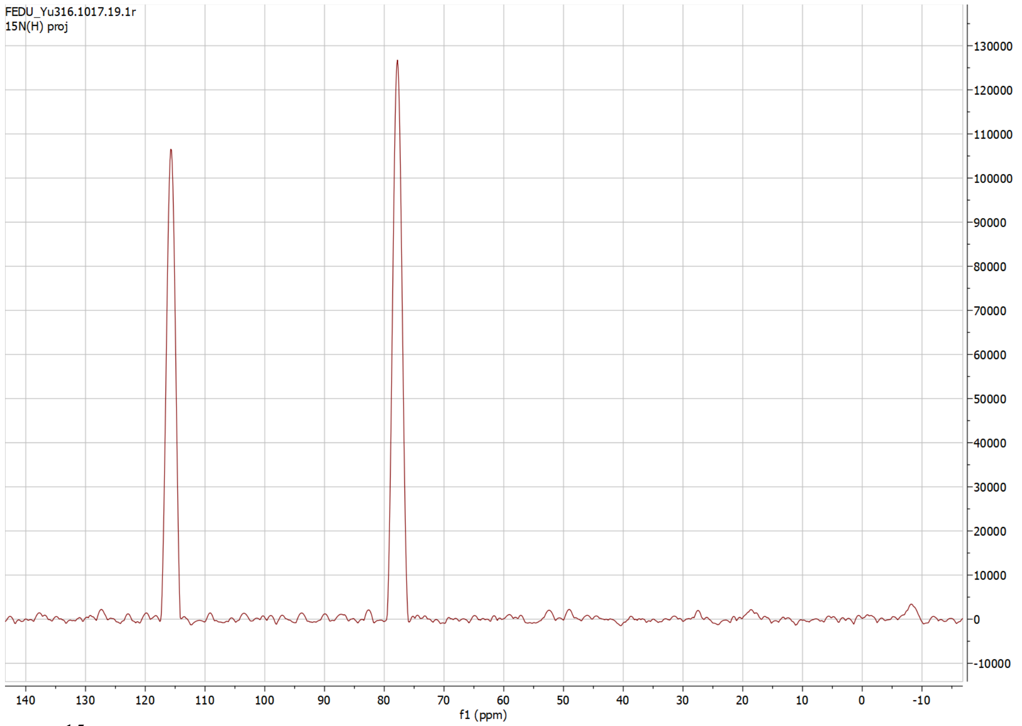

Figure S17. ^{15}N NMR spectrum of 3-amino-10-(3,5-dichloro-2-hydroxyphenyl)-1-hydroxy-7,7-dimethyl-9-oxo-5,6,7,8,9,10-hexahydrobenzo[*b*][1,6]naphthyridine-4-carbonitrile **3a** in DMSO-d_6.

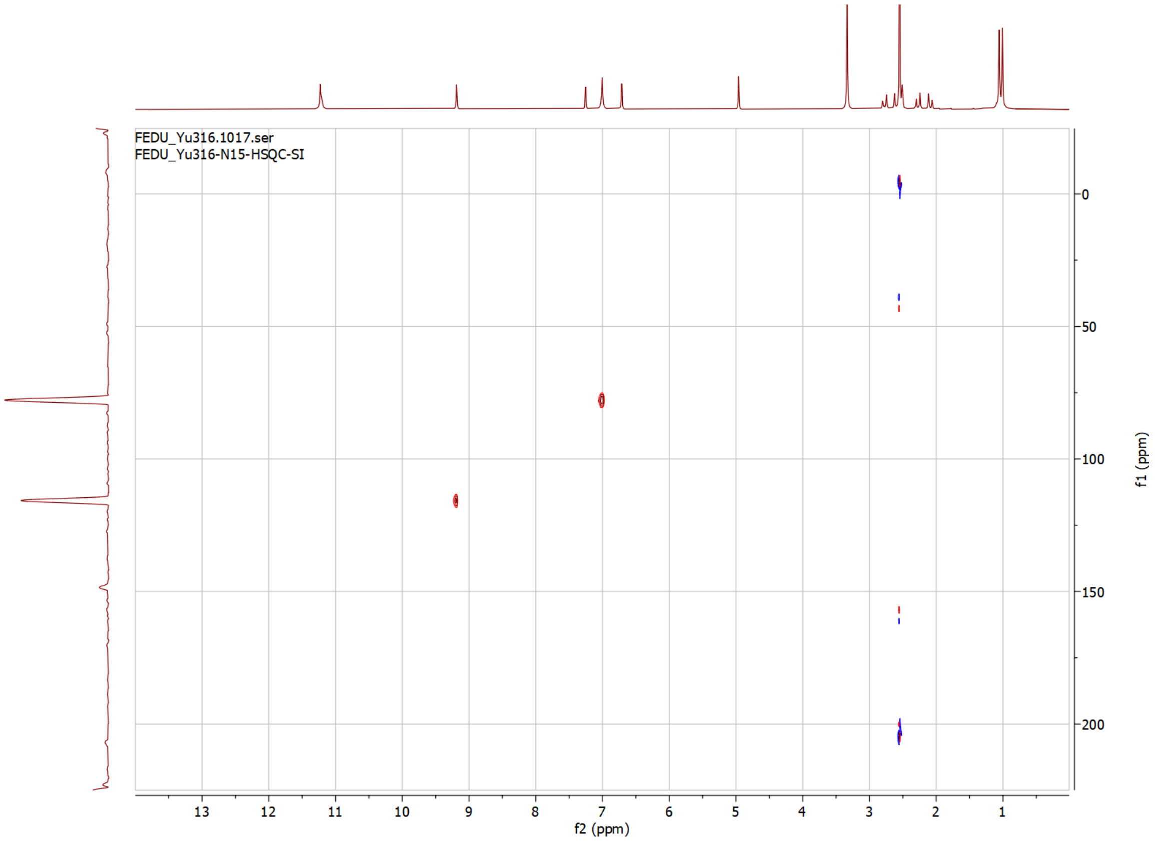

Figure S18. ^{1}H-^{15}N HSQC NMR spectrum of 3-amino-10-(3,5-dichloro-2-hydroxyphenyl)-1-hydroxy-7,7-dimethyl-9-oxo-5,6,7,8,9,10-hexahydrobenzo[*b*][1,6]naphthyridine-4-carbonitrile **3a** in DMSO-d_6.

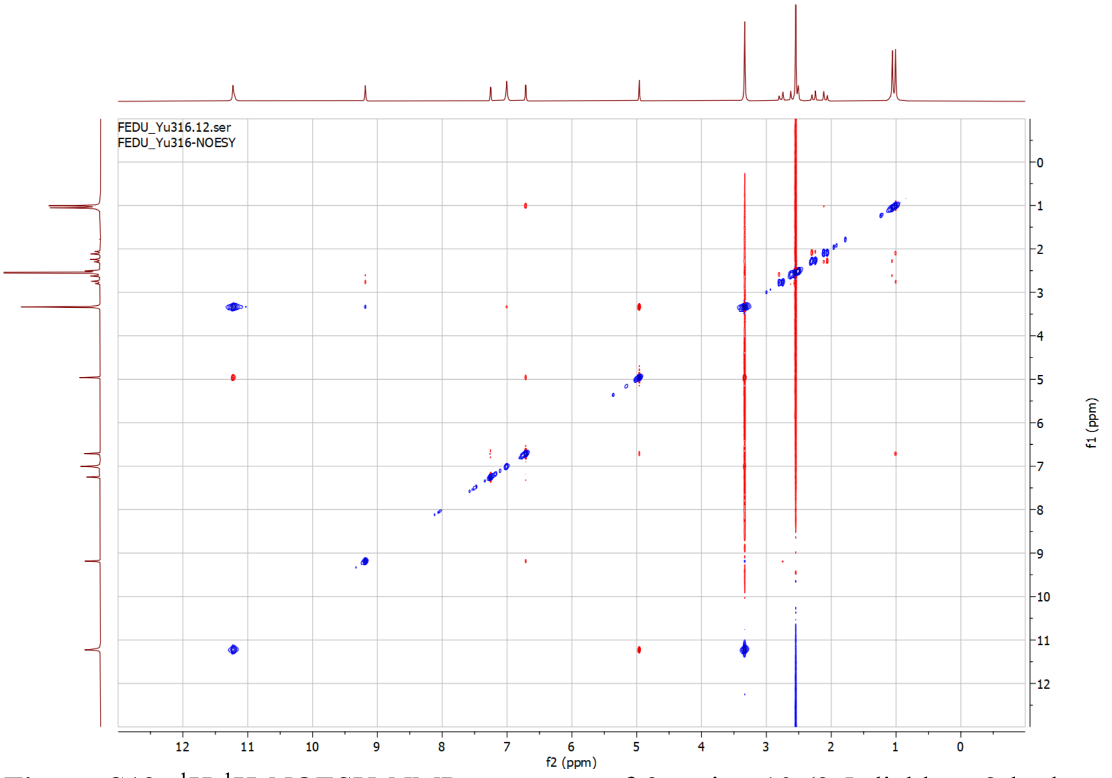

Figure S19. ^{1}H-^{1}H NOESY NMR spectrum of 3-amino-10-(3,5-dichloro-2-hydroxyphenyl)-1-hydroxy-7,7-dimethyl-9-oxo-5,6,7,8,9,10-hexahydrobenzo[*b*][1,6]naphthyridine-4-carbonitrile **3a** in DMSO-d_6.

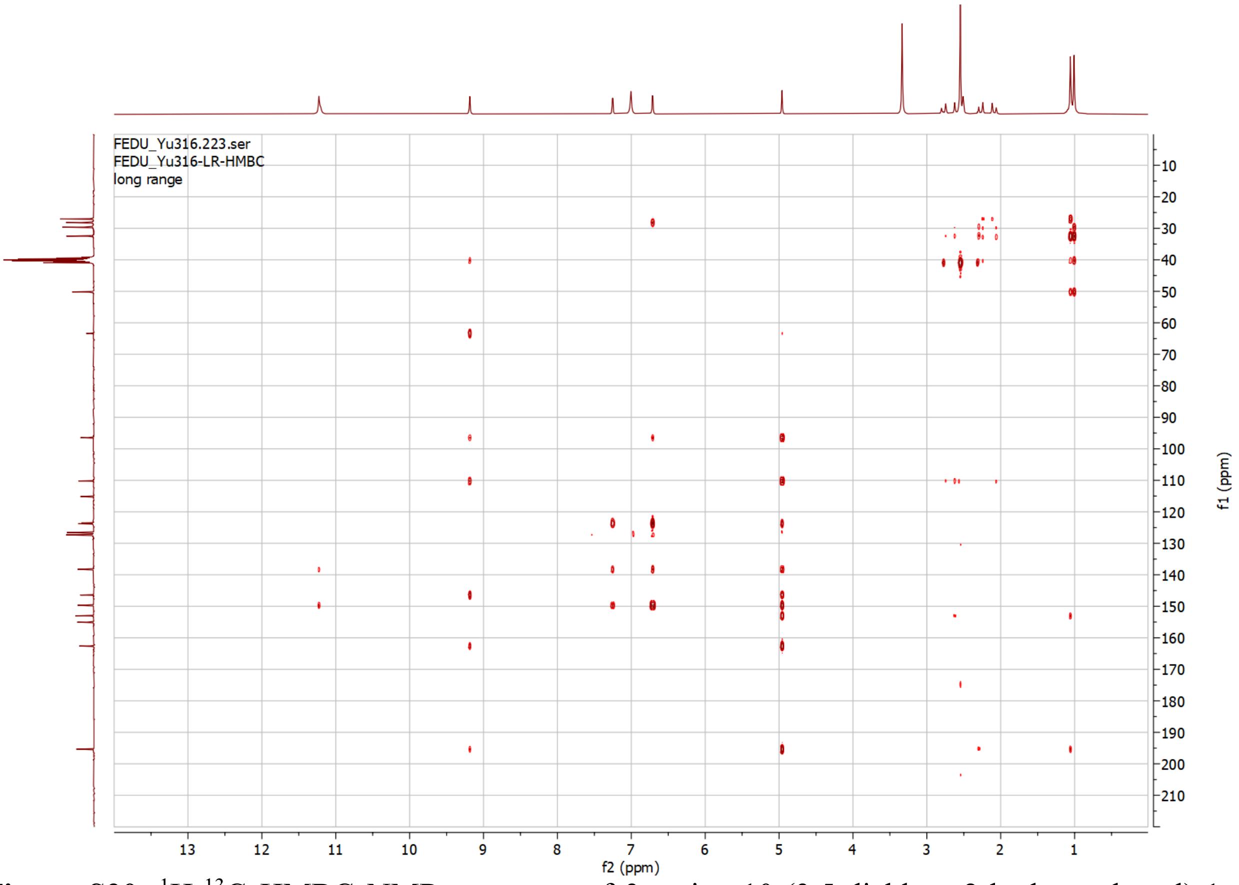

Figure S20. ^{1}H-^{13}C HMBC NMR spectrum of 3-amino-10-(3,5-dichloro-2-hydroxyphenyl)-1-hydroxy-7,7-dimethyl-9-oxo-5,6,7,8,9,10-hexahydrobenzo[*b*][1,6]naphthyridine-4-carbonitrile **3a 2a** in DMSO-d_6.
